# Supplementary material for: The involvement of non-governmental organisations in achieving health system goals based on the WHO six building blocks: A scoping review on global evidence
Source: PLoS One. 2025 Jan 30;20(1):e0315592. doi: 10.1371/journal.pone.0315592 (PMC11781716; doi:10.1371/journal.pone.0315592)
Supplement: S2 Table — (DOCX) [file pone.0315592.s002.docx]

S 2 Table: Search strategies

| Databases | Search strategy |
| --- | --- |
| EMBASE | ,"('health service'/de OR 'health care agency':ti,ab,kw OR 'health care service':ti,ab,kw OR 'health maintenance service':ti,ab,kw OR 'health practice':ti,ab,kw OR 'health service':ti,ab,kw OR 'health services':ti,ab,kw OR 'health services administration':ti,ab,kw OR 'health services for persons with disabilities':ti,ab,kw OR 'health services for transgender persons':ti,ab,kw OR 'health services for transgendered persons':ti,ab,kw OR 'health services misuse':ti,ab,kw OR 'health services needs and demand':ti,ab,kw OR 'health system agency':ti,ab,kw OR 'health visiting':ti,ab,kw OR 'healthcare agency':ti,ab,kw OR 'healthcare service':ti,ab,kw OR 'medical health service':ti,ab,kw OR 'menu planning':ti,ab,kw OR 'personal health services':ti,ab,kw OR 'physician service':ti,ab,kw OR 'reproductive health services':ti,ab,kw OR 'service, health':ti,ab,kw OR 'student health care':ti,ab,kw OR 'student health service':ti,ab,kw OR 'student health services':ti,ab,kw OR 'suburban health services':ti,ab,kw OR 'tuberculosis societies':ti,ab,kw OR 'urban health services':ti,ab,kw OR 'voluntary health agencies':ti,ab,kw OR 'women`s health services':ti,ab,kw) AND ('non-governmental organization'/de OR 'ngo (non government organization)':ti,ab,kw OR 'ngos (non government organizations)':ti,ab,kw OR 'non government organization':ti,ab,kw OR 'non governmental organization':ti,ab,kw OR 'non-government organisation':ti,ab,kw OR 'non-governmental organisation':ti,ab,kw OR 'non-governmental organization':ti,ab,kw OR 'nongovernment organisation':ti,ab,kw OR 'nongovernment organization':ti,ab,kw OR 'nongovernmental organisation':ti,ab,kw OR 'nongovernmental organization':ti,ab,kw OR 'non-government sector':ti,ab OR 'non state provider*':ti,ab)",656,10 Jan 2024 |
| Scopus: January 02, 2024 | ( TITLE ( "non-government sector" ) OR TITLE ( "non-governmental organization" ) OR TITLE ( ngo ) OR TITLE ( "non-state providers" ) AND TITLE ( "health service" ) OR TITLE ( "health system" ) OR TITLE ( "health service delivery" ) OR TITLE ( "service coverage" ) OR TITLE ( "health workforce" ) OR TITLE ( leadership ) OR TITLE ( governance ) OR TITLE ( information ) OR TITLE ( "medical products" ) OR TITLE ( vaccines ) OR TITLE ( management ) OR TITLE ( financing ) OR TITLE ( financial ) OR TITLE ( stewardship ) OR TITLE ( "health technology" ) OR TITLE ( equipment ) ) AND PUBYEAR > 1999 AND PUBYEAR < 2024 AND ( LIMIT-TO ( DOCTYPE , "ar" ) OR LIMIT-TO ( DOCTYPE , "re" ) ) |
| Web of Science: January 02, 2024 | (((TI=(non-government sector )) OR TI=(non-governmental organization )) OR TI=(NGO*)) OR TI=(non-state provider*) AND (((((((((((((((TI=(health service)) OR TI=(health system)) OR TI=(health service delivery)) OR TI=(service coverage)) OR TI=(health workforce)) OR TI=(Leadership)) OR TI=(governance)) OR TI=(Information)) OR TI=(medical products)) OR TI=(vaccines)) OR TI=(management)) OR TI=(Financing)) OR TI=(financial)) OR TI=(stewardship)) OR TI=(health technology)) OR TI=(equipment) |
| PubMed | ("non governmental organization"[Text Word] OR "non government organization"[Text Word] OR "nongovernment organisation"[Text Word] OR "nongovernment organization"[Text Word] OR "nongovernmental organisation"[Text Word] OR "non government sector"[Text Word] OR "non state provider*"[Text Word]) AND (("Health Services"[Majr]) OR ('health service'[Text Word] OR 'health care agency'[Text Word] OR 'health care service'[Text Word] OR 'health maintenance service'[Text Word] OR 'health practice'[Text Word] OR 'health service'[Text Word] OR 'health services'[Text Word] OR 'health services administration'[Text Word] OR 'health services for persons with disabilities'[Text Word] OR 'health services for transgender persons'[Text Word] OR 'health services for transgendered persons'[Text Word] OR 'health services misuse'[Text Word] OR 'health services needs[Text Word] AND demand'[Text Word] OR 'health system agency'[Text Word] OR 'health visiting'[Text Word] OR 'healthcare agency'[Text Word] OR 'healthcare service'[Text Word] OR 'medical health service'[Text Word] OR 'menu planning'[Text Word] OR 'personal health services'[Text Word] OR 'physician service'[Text Word] OR 'reproductive health services'[Text Word] OR 'service, health'[Text Word] OR 'student health care'[Text Word] OR 'student health service'[Text Word] OR 'student health services'[Text Word] OR 'suburban health services'[Text Word] OR 'tuberculosis societies'[Text Word] OR 'urban health services'[Text Word] OR 'voluntary health agencies'[Text Word] OR 'women`s health services'[Text Word])) |
