# Supplementary material for: The involvement of non-governmental organisations in achieving health system goals based on the WHO six building blocks: A scoping review on global evidence
Source: PLoS One. 2025 Jan 30;20(1):e0315592. doi: 10.1371/journal.pone.0315592 (PMC11781716; doi:10.1371/journal.pone.0315592)
Supplement: S3 Table — (DOCX) [file pone.0315592.s003.docx]

**S 3 Table. Characteristics of included studies**

| **First Author/ year** | **Country/ type** | **Study design** | **aim of study** | **Levels of care** | **Population coverage** | **Intervention(s)** | **Implementation considerations** |
| --- | --- | --- | --- | --- | --- | --- | --- |
| Yagub., et al., 2015 **(46)** | Sudan/ LI | Mix method | Exploring difficulties that faced the collaboration between the government and NGOs sectors and their impact on curative health service delivery | Primary care | Vulnerable people | Providing health services through the provision of human resources, health facilities and health materials and equipment | The need for a new collaboration model between governments and NGOs based on mutual trust is felt. |
| Albis et al., 2019  **(47)** | Bangladesh/ LMI | Survey | Evaluation impact of the second phase project (UPHCP-II) on health outcomes | Primary care | Poor urban communities | Providing Primary Health Care | NGOs should be considered as partners in government primary health care service programs. |
| ‏Amirkhanian et al., 2004  **(48)** | Central and Eastern Europe | Qualitative study | Exploring programs, resources and challenges of AIDS prevention NGOs | Primary care | People with AIDS | Providing prevention, education and services | NM |
| Ejaz, I.  **(50)** | Pakistan, 2011/ LMI | Qualitative study | Exploring role and position of NGOs in health systems strengthening in Pakistan's context. | Primary care | Community of Pakistan | Provide health education, health promotion and health services | The expansion of trust and support from the government is vital for NGOs to efficiently execute their duties. |
| Mercer, M. A.  **(51)** | Timor-Leste, 2014/ LMI | Qualitative study | Exploring role of NGOs International in strengthening health systems | Primary care | Traumatized population | Providing health services | NM |
| Wamai, R. G  **(52)** | Kenya and Ethiopia, 2008/ LMI, LI | Qualitative study | Reforming health system and the role of NGOs in decentralization | Primary care | Urban areas | providing public and health services (treatment, prevention and rehabilitation), HIV / AIDS and reproductive health services | NM |
| Mercer, A  (53) | Bangladesh, 2004/ LMI | Review | This paper considers evidence of the effectiveness of a non-governmental organization (NGO) primary health care program in rural Bangladesh. | Primary care | Poorest people | Providing clinical education, vaccination, reproductive health and child health services | The development of health plans necessitates a participatory process that actively engages local stakeholders in planning process. |
| Mercer, A  (54) | Bangladesh, 2006/ LMI | Survey | Aiming of the present study is to validate the recording of neonatal deaths | Primary care | Rural areas among the poor and needy in 12 areas | Providing reproductive health services | NM |
| De Maio, G.  (55) | Italy, 2014/ HI | Descriptive study | Aiming to document the characteristics and clinical management of inpatients and outpatients for homeless. | Primary care | Homeless | Providing inpatient and outpatient services | NM |
| De Souza, R.  (56) | India, 2009/ LMI | Qualitative study | The processes used by a local nongovernmental organization called the Society for People's Action for Development to organize sex workers in the slums of Bangalore, India, for HIV/AIDS prevention. | Primary care | Slums of Bangalore, India, | Organising sex workers for HIV/AIDS prevention | NM |
| Dhingra, R.  (57) | India, 2001/ LMI | Qualitative study | Exploring the factors associated with the long-term success of Community - based health insurance. | Primary care  Secondary care | Indian population | Financing the provision of preventive and curative services through community-based health insurance programs | NGOs can promote social health insurance programs in developing countries. |
| Franco, M. M. R.  (58) | Mexico, 2018/ UMI | Quantitative retrospective analysis | Describing experience of a tertiary/referral center in Mexico City | Tertiary care | Vulnerable patients | Providing pharmaceutical and chemical subsidies to hematopoietic cell transplant patients | NGOs can be successful in making health changes for specific diseases. |
| Ghosh, S. C.  (60) | Bangladesh, 2011/ LMI | Mix method | Exploring of NGOs in improving sanitation status in the rural areas of Bangladesh | Primary care | Rural areas | Establishing toilets to improve their health. | NGOs play a vital role in improving the health of people in rural areas. |
| Gomez-Jauregui, J.  **(62)** | Mexico, 2004/ UMI | Qualitative study | Analyzing whether the conditions exist in Mexico for successful partnerships between the public sector and NGOs in the reproductive health field | Primary care | Rural area | Providing reproductive health services | NM |
| Heard, A  **(63)** | India, 2011/ LMI | Mix method | Assessing the large-scale contracting of 294 non-governmental organizations (NGOs) | Primary care | Remote areas | Providing of basic health services, reproductive health, basic child health care, and health education | NM |
| Holland, C. E.  (64) | Cameroon, 2015/ LMI | Cross-sectional survey | Aiming to examine the uptake of HIV services at non-governmental organizations | Primary care | Men who have sex with men (MSM) | Providing AIDS/HIV services from NGOs | NM |
| Khan, J. A.  (65) | Bangladesh, 2017/ LMI | Survey | Investigating the extent to which health benefits are distributed across socioeconomic groups | Primary care | Rural area | Providing services to mothers, infants and children, prevention and health care | NM |
| Khodayari-Zarnaq, R.  **(66)** | Iran, 2019/ UMI | Qualitative study | Aiming to effectiveness of non-governmental organization (NGO) participation in the healthcare sector | Primary care | Vulnerable and poor groups | Establishing and equipping hospitals, supplying medicine and treatment to the poor, financial support for orphans and other people, providing loans, awareness, and training | Realizing the potential of NGOs could be accomplished with government financial support and the implementation of supportive laws. |
| Manna, A.  (68) | India, 2019/ LMI | Qualitative study | Exploring whether communication by mobile phone can lessen burden cancer patients | Tertiary care | Cancer patients | Using cell phones to communicate | NM |
| Mehta, P.  **(69)** | India, 2013/ LMI | Cross-sectional | Evaluation the improvement in cancer survival in pediatric patients through funding for person-ell by a nongovernmental | Tertiary care | Children with cancers | Providing medical service | It is important to collaboration with NGOs to improve the provision of services in developing countries |
| Momoh, G. T.  (70) | Nigeria, 2015/ LMI | Quasi experimental | Aiming strengthening the capacity of 12 non-governmental organizations on advocacy and policy related activities with emphasis on reproductive health issues | Primary care | Women | Strengthen the capacity of 12 NGOs in the field of support and policy related to the emphasis on reproductive health issues | NM |
| Mugisha F  (71) | Uganda, 2005/ LI | Qualitative study | Exploring ability for reproductive health (RH) non‐governmental organizations (NGO) in Uganda | Primary care | Women | Providing reproductive health | Decentralization in the government and outsourcing some activities to NGOs will strengthen both sides. |
| Mukherjee S  (72) | India, 2017/ LMI | Descriptive study | Examining discriminatory trend in existing Government health care structure in  Bangalore city | Primary care | Women | Community empowerment for Health Promotion | Strengthening community capacity and NGOs in the form of community mobilization can help in improving awareness, demand and utilization of health services. |
| Nguyen, N.  (73) | LMI, 2014 | Online survey | Surveying NGOs Providing Pediatric Cardiovascular Care in Low- and Middle-Income Countries | Secondary care | Children | Providing clinical services to cardiovascular programs | NM |
| Perry, H.  (74) | 28 countries such as  Bangladesh  BoLIvia  Burkina Faso  Burundi  Cambodia, 2015/ LI, LMI, UMI, HI and Fragile | Qualitative study | Describing Innovative Community-Based Strategy for Improving Maternal, Neonatal, and Child Health in Resource-Constrained Settings approach | Primary care | Women and children | Providing health of mothers, neonates, and children, and empowering women | NM |
| Perry, H.  (75) | 28 countries such as  Bangladesh  BoLIvia  Burkina Faso  Burundi  Cambodia, 2015/ LI, LMI, UMI, HI and Fragile | Qualitative study | Summarizing available evidence on the effectiveness of the Care Group approach | Primary care | Women and children | Providing health of mothers, neonates, and children, and empowering women | NM |
| Piotrowicz M  (76) | Poland, 2013/ HI | Qualitative study | Aiming of definitions, objectives, fields and tasks of NGOs in social life, health system and health policy | Primary care | Community | Providing services and health advocacy | NM |
| Ricca, J.  (77) | Sub-Saharan Africa, South and Southeast Asia and the Caribbean, 2014 | Mix method | Aiming at testing the generalizability of the published findings on the mortality | Primary care | Children | NGO projects implementing community-based intervention packages to child mortality | NGO-facilitated projects utilizing community-based intervention packages can contribute to improving coverage for multiple high-impact interventions simultaneously at the scale of one or several districts in countries. |
| Ui, S.  (78) | Cambodia, 2010/ UMI | Descriptive study | Identifying factors facilitating community participation in health center management | Primary care | Rural area | Community participation at health centers | NM |
| Abdelmoneium, A. O. A.  (80) | Sudan, 2010/ LI | Qualitative study | Focusing on the work of an international organisation working in health services | Primary care | Adolescent mothers | Providing for separate provision of services | NGOs should adopt a rights-based approach which encompasses equality in both gender and health services. |
| Ahmed, N.  (81) | Sudan, 2019/ LI | Qualitative study | Describing the private sector contributions to equitable access to immunisation services and coverage | Primary care | Children | Providing free vaccinations | NGOs participation is very important in equitable access to immunization services and coverage. |
| Kelly, Jeffrey A.  (82) | Africa, Central/Eastern Europe and Central Asia, Latin America and the Caribbean, 2006 | Qualitative study | This study assessed the programmes, resources, and needs of HIV-prevention nongovernmental organizations | Primary care | Population with HIV/AIDS | African NGOs most likely to use peer education and community awareness events; Eastern European NGOs most likely to offer needle exchange; Latin American NGOs to have resource centres and offer risk reduction programs; and Caribbean organisations to use mass education approaches | NM |
| Ambrosini, M.  (84) | Italy, 2015/ HI | Case studies | Describing the results of two case studies on NGOs (NEGA and OSF) based in Milan | Primary care | Irregular immigrants | Providing free health care | The collaboration of the government with NGOs can be effective on policies to reduce cost-cutting on social exclusion. |
| Ament, J. D.  (85) | Bolivia, 2014/ LMI | Cross-sectional | Determining the impact and cost-effectiveness of an NGO intervention | Tertiary care | Patients with spinal pain | Support for spinal procedures | NGOs can be cost-effective with effective interventions in special diseases with high costs. |
| Andrade, M.  (86) | Brazil, 2018/ UMI | Non-randomised trial | Evaluating the effectiveness of the assistance provided by both NGOs | Tertiary care | Patients with cancer | Providing diagnostic mammography and biopsies as well as anatomo-histopathological and immunohistochemical analysis | NGOs' mediation can significantly improve patient flow. |
| Bader, F.  (87) | Jordan, 2009/ UMI | Survey | Exploring the factors associated with a need for psychological services among patients at seven clinics served | Primary care | Displaced population | Providing mental health | NM |
| Baig, M. B.  (88) | India, 2014/ LMI | mixed method | Aiming to understand the breadth and depth of services in primary health | Primary care | Population of Kendrapara district of Odisha | Providing primary health centres and primary health care | Critical factors for enhancing intersectional collaboration include having partners who are both strong, as well as being embedded in policy environments that offer support. |
| Baqui, A. H.  (89) | India, 2008/ LMI | Quasi-experimental study | Examining whether NGO facilitation of the government's community-based health programme | Primary care | Children | Providing of maternal and newborn health | NM |
| Barzin, Y.  (90) | Vietnam, 2012/ LMI | Qualitative study | Investigating of a NGO's medical programme, which provides free temporary clinics | Primary care | Rural Vietnam | Providing health services | NGO programs in the field of health do not seem to undermine the public health system. |
| Cancedda, C.  (91) | Sierra Leone, 2016/ Fragile | Qualitative study | Describing how the international NGO Partners In Health (PIH) partnered with the Government | Primary care | Ebola virus disease patients | Provide 17 health facilities in 4 regions and to establish 2 laboratories and employee 800 CHW for fight Ebola virus disease | NM |
| Chanani, S.  (92) | India, 2019/ LMI | Cross-sectional | Describing performance of a community-based nutrition programme in preventing | Primary care | Children in urban India | Providing prevention and treatment groups received growth monitoring, referrals to public health facilities, and home‐based counselling | To promote better coverage, health recovery outcomes, and more efforts to create community awareness, ownership and involvement of NGOs in prevention and treatment activities are needed. |
| Devadasan, N.  (93) | India, 2012/ LMI | Cross-sectional survey | Surveying of evidence of improved health care with access to the quality of access and prevention of poverty through community health insurance (CHI) program. | Primary care | Poor people | Insuring poor people through NGOs | A well-designed community health insurance (CHI) program can able to increase the access to health care and reduce OOP payments. |
| Edward, A.  (94) | Afghanistan, 2015/ Fragile | Mixed-method | Conducting capacity assessments of the community health worker (CHW) | Primary care | Women | NGOs provide comprehensive training for CHWs | NM |
| Ferguson, J. L.  (95) | Australia, 2018 / HI | Qualitative study | Describing the aims and organizational structures and responsibilities of each service | Primary care | Poor patients | Providing facilitating diversion from hospitalisation (step-up) and providing residential support services following discharge from the hospital (step down). | Collaboration between the government and NGOs is important and necessary. |
| Fiorini, G.  (96) | Italy, 2016/ HI | Cross-sectional | Describing insight into the prevalence of chronic noncommunicable diseases (CNCD) in undocumented migrants | Primary care | Undocumented migrants in Milan | Providing health services for chronic noncommunicable diseases, drug dispensation, and providing free medical assistance | NM |
| Gilbert, H.  (97) | Mozambique and Kenya, 2011/ LI And LMI | Qualitative study | Examines and compares the role and effectiveness of two separate NGOs involved since 2001 in HIV/AIDS management programmes | Primary care | Population with HIV/AIDS | Providing HIV/AIDS awareness, HIV/AIDS prevention, access to HIV healthcare services and the provision of treatment | NM |
| Heinmüller, R.  (98) | Mali, 2012/ LI | Time series | Impacting on health center attendance by children under five. | Primary care | Children under-fives with malaria | Dispensing free care to under-fives for cases of malaria that covered a rapid diagnostic test (RDT) and a course of artemisinine-based combination therapy (ACT) through Medicines Sans Frontiers | NM |
| Huff-Rousselle, M.  (99) | Cambodia, 2001/ LMI | Survey | Examining and comparing aspects major government and the major indigenous non-government clinics | Primary care | Women | Provision of reproductive health services by an NGO clinic | NM |
| Mahyiuob Al-Honahi, H. Y.  (100) | Yemen, 2010/ Fragile | Descriptive Observational study | Show the feasibility of involvement of Non-Governmental Organization (NGO) health volunteers with regular monitoring mechanism on tuberculosis (TB) control | Primary care | Ebola virus disease patients | Finding and case holding activities by the national tuberculosis control program staff and involvement health volunteers for intervention tuberculosis | NM |
| ‏Matousek, A. C.  (101) | Haiti, 2015/ LI | Cross-sectional | Analyzing the equity achieved under the financial system at both hospitals | Tertiary care | Poorest rural Haiti | Providing equitable surgical care through free care by 2 NGO hospital | Health systems striving to serve the poor should continually evaluate and seek to improve equity, even within systems that provide free care. |
| Mukherjee, J. S.  (102) | Haiti, 2007/ LI | Mixed method | Describing the contribution of the non-governmental organization, to the HIV prevention | Primary care | Population with HIV/AIDS | Zanmi Lasante (NGO) has recruited, trained and financed a large cadre of CHWs to provide such linkages between communities and health centres | NM |
| Nunns, D.  (103) | Nepal, 2011/ LMI | Country case study | Describing the Working with UK‐based non‐governmental organisations (International Nepal Fellowship) for better reproductive health in Nepal | Primary care | Women | Providing reproductive health care | NGOs and donors have played an important role in health care in LMI countries over the past decades and continue to provide high quality care in regions poor rural. |
| Odindo, M. A.  (104) | Kenya, 2008/ LMI | Descriptive cross-sectional | Assessing the role of governmental and non-governmental organizations in mitigation of stigma and discrimination among people | Primary care | Orphans with HIV/AIDS | Providing awareness, outreach, counselling, testing, treatment, advocacy, home-based care, assistance to the orphans and legal issues. | NM |
| Oleribe, O. O.  (105) | Nigeria, 2018/ LMI | Cross-sectional | Reporting to document the process and outcome of practice "Test and Start Strategy" | Primary care | Population with HIV/AIDS | Providing HIV testing and counselling, disclosure of results, post-test counselling and healthy lifestyle education and, distribution of free male condoms and Information, Education and Communication (IEC) material | NM |
| Oseji, M  (106) | Nigeria, 2014/ LMI | Review | Examine the contributions of professional health associations, non-governmental organisations, government ministries, and community-based organisations in implementing community-based interventions for the reduction of maternal mortality in Delta State Nigeria. | Primary care | Women | Providing advocacy, awareness creation, and sensitisation programmers on reproductive health using behaviour change communication materials. | NM |
| Ridde, V.  (107) | Burkina Faso, 2012/ LI | Qualitative study | Investigating how NGOs selected the beneficiaries of payment exemptions for government-provided antiretroviral treatment (ART) | Primary care | Population with HIV/AIDS | Providing free HIV treatment and services | NM |
| Sankaran, S.  (109) | India, 2017/ LMI | Qualitative study | Describing the development of a program that trained CHWs between 2014 and 2015 | Primary care | Community | Training CHWs to screen for and manage chronic hypertension | NM |
| Sarwar, M. R.  (110) | Bangladesh, 2015/ LMI | Case studies | Examining the factors contributing to successful partnerships for health market | Primary care | Children | Providing maternal and child health and distribution of a micronutrient food supplement | NM |
| Sharma, A. K  (111) | India, 2010/ LMI | Randomised control trial | Impacting of wholesome mid-day meal (MDM) program run by an NGO | Primary care | Children | Providing mid-day meal for primary school students | NM |
| Singh, M. M  (112) | India, 2015/ LMI | Descriptive | Describing 100 People living with HIV/AIDS (PLHAs) admitted at 3 care homes run by nongovernmental organizations in New Delhi | Primary care | People living with HIV/AIDS | Providing care home for PLHA | NM |
| Singh, V.  (113) | India, 2017/ LMI | Quasi-experimental | Evaluating the effectiveness of enhancing a nutrition and health program on breastfeeding and complementary-feeding practices | Primary care | Women | Providing services delivered by community-based nutrition and health care providers | NM |
| Sivakumar, T.  (114) | India, 2019/ LMI | Cross-sectional descriptive | Describing impact of Community Based Rehabilitation (CBR) on OOP expenditure incurred by families | Primary care | Persons with severe mental illness | Providing mental health services through Community Based Rehabilitation (CBR) | The participation of the public health and NGOs leads to a significant reduction in the OOP health expenditure of families. |
| Soe, K. T  (115) | Myanmar, 2017/ Fragile | Cross-sectional descriptive | Describing four INGO strategies for providing community-based TB care | Primary care | TB disease patients | Providing community-based TB care to hard-to-reach populations | NM |
| Solomon, Y.  (116) | Mali, 2008/ LI | Mix method | Examining the experiences of an NGO-sponsored health care program | Primary care | Rural area | Providing primary health care | NGOs facilitate community participation in a decentralized decision-making and dialogue process. |
| Thomas, R.  (117) | India, 2013/ LMI | Cross-sectional descriptive | Investigating the prevalence of malnutrition using anthropometric measures in a cohort of tribal students | Primary care | Students | Providing three meals a day of tribal students attending a school in rural south India. | NM |
| van de Vijver, S.  (118) | Kenya, 2013/ LMI | Qualitative study | Developing and introducing a model of cardiovascular by integrating public health and private sector approaches | Primary care | Slums of Nairobi | Providing cardiovascular prevention | NM |
| Wandwalo, E.  (119) | Tanzania, 2004/ LMI | Mix method | Determining the feasibility of establishing collaboration between the tuberculosis programme and an NGO in TB/ HIV care at a district level in Tanzania | Primary care | Population with HIV/AIDS | Providing voluntary counselling and testing for HIV, diagnosis and treatment of TB, referral and follow up of patients and suspects, home-based care, psychological support and training | Effective government programs require the inclusion and active involvement of NGOs as valued partners. |
| Zachariah, R.  (120) | Malawi, 2004/ LMI | Qualitative study | Non - governmental organizations role in implementing joint TB and HIV interventions in rural Malawi. | Primary care | Population with HIV/AIDS | Providing additional staff, supplementary drugs including antiretroviral drugs, technical assistance and infrastructure development | Participation and presence of NGOs, a new dynamic of decentralized operational flexibility that improves access to care and support for health services. |
| Danni, W.  (121) | China, 2016/ UMI | Cross-sectional | Providing evidence to policy makers, this paper describes the basic information on NGOs and their shortage of social capital. | Primary care | Population with HIV/AIDS | Providing HIV/AIDS prevention and control in China | As an emerging topic in international academic research, social capital can offer a new perspective to promote the development of NGOs. |
| Anna, B.  (122) | Spain, 2011/ HI | Mixed method | Describing and analyzing the objectives and HIV-AIDS preventive activities | Primary care | Population with HIV/AIDS | Providing HIV/AIDS prevention and control in Catalonia | NGOs will be able to implement the strategies we need to keep in mind and strengthen future prevention programs. |
| Zihindula, G.  (123) | South Africa, 2019/ UMI | Review | Conducting to ascertain the role NGOs in addressing the shortage of healthcare professionals in rural South Africa |  | Rural South Africa | Providing healthcare professionals | NGOs have a crucial role to play in the training-placement and retention of healthcare professionals and that their contributions can make a difference in the health sector if approved and recognized. |
| Sarriot et al., (124) | 2004 | Qualitative study | Describing the Child Survival Sustainability Assessment (CSSA) methodology | Primary care | Children | Providing child health interventions | NM |
| Biermann, O.  (125) | Ecuador 2016/ UMI | Qualitative study | Exploring how an NGO and its health services are perceived by the population that it serves, and how it can contribute to reducing barriers to care | Primary care | Community | Providing health services | Public private partnership (PPP) parties should develop a common platform with joint messages to the target population on the provider’s structure, and regarding partners’ roles and responsibilities |
| Arman, S.  (126) | 2020 | Scoping review | Reviewing the role of NGOs and their engagement strategies in progress toward UHC. | Primary care  Secondary care  Tertiary care | Community | NGOs providing service, financial, and population coverage for community | The government should use strategies and interventions in supporting NGOs. |
| Yoon, A. S.  (127) | Sierra Leone 2018/LI | Qualitative study | Aiming to examine the effectiveness of INGOs in a context of managing a fatal epidemic outbreak of Ebola | Primary care | Ebola disease patients | NGOs in Sierra Leone, building healthcare infrastructures, providing medical supplies, educating local residents, and training response staffs. | policy tools, which allow INGOs to enter to the field in a timely manner, can improve the effectiveness of INGOs responses in current and future epidemic outbreaks in developing countries where people suffer from a lack of health infrastructures. |
| Monir, B. E.  (128) | Iran 2014/  UMI | Qualitative Study | Understanding the nature of participation practice in Community based health program and to use the data to advocate for more participation friendly policies in the community, academy and funding organizations | Primary care | Community | NGOs in Iran providing Community based health programs. | For intersectional collaboration, strong and dedicated partners, supportive policy environment are critical. |
| Sanadgol, A.  (129) | Iran 2022/  UMI | Qualitative Study | Investigating the viewpoints of Iranian health system experts and executive stakeholders on the role of NGOs in moving toward UHC | Primary care  Secondary care  Tertiary care | Community | NGOs providing service, financial, and population coverage for community in Iran | Recognizing the critical role of NGOs and their contribution is essential, particularly in the local context. Collaboration between NGO stakeholders and the government could facilitate achieve of goals health system. |
| Helen, K.  (130) | England 2015/ UMI | Review | Answering the question, ‘to what extent can evaluation frameworks help NGOs to address health inequalities caused by social exclusion? | Primary care | Community | NGOs providing public health services in England | NGOs should place social value at the centre of the evaluation process, or at least make sure they capture as much as they can of the social value they create, in line with their own value base |
| Paul A. B.  (131) | USA 2020/  HI | Qualitative study | Describing two case studies presented were implemented by community-based organizations | Primary care | Population with HIV/AIDS | Providing HIV prevention and health-care needs of Black men who have sex with men |  |
| Lim, S.  (132) | Kenya and Zambia 2022/ LMI, LI | Survey | Investigating whether development projects run by international NGOs are able to fill this gap and help children to remain hopeful during the pandemic | Primary care | Children | Providing children’s mental health |  |
| Sanadgol, A.  (133) | Iran 2022/  UMI | Qualitative Study | Exploring effective strategies for NGO participation in the Iranian health system to achieve broader health system goals. | Primary care  Secondary care  Tertiary care | Community | NGOs providing service, financial, and population coverage for community in Iran | All elements and dimensions of this process need to be considered when developing a platform for the appropriate participation of NGOs in the health system functions. Evidence-informed strategies for strengthening the participation of NGOs in the health system should be used to utilize NGOs potential to the fullest. |
| Aradhana, S.  (134) | India 2016/ LMI | Qualitative Study | Exploring level and types of linkages between public health sector and NGOs | Primary care | Women and children | Providing Maternal and child health | For sustainable and systematic cooperation, with joint planning, implementation and evaluation, NGOs and the government need stronger ties. |
| [Babis](https://scholar.google.com/citations?user=pM5tQOoAAAAJ&hl=en&oi=sra), B.  (135) | Bolivia 2014/ LMI | qualitative | Tracing the constellation of forces that led to the institutionalization of indigenous medicine | Primary care | Indigenous doctors and leaders | Indigenous doctor organizations in Bolivia have been highly involved throughout the entire process of institutionalization |  |
| Sajadi H. S.  (136) |  | Scoping review | Identifying options proposed or implemented to improve the participation of the NGOs in preventive care | Primary care | Community | NGOs providing health care | Information gap in the effect of interventions to improve NGOs' participation in health |
| I. Sikazwe, C.  (137) | Zambia 2023/ LI | Qualitative study | Recognizing of the unique role that local NGOs bring to the HIV response | Primary care | Population with HIV/AIDS | Providing integrate, and scale up evidence-based practices for HIV prevention, care, and treatment |  |
