# Supplementary material for: The involvement of non-governmental organisations in achieving health system goals based on the WHO six building blocks: A scoping review on global evidence
Source: PLoS One. 2025 Jan 30;20(1):e0315592. doi: 10.1371/journal.pone.0315592 (PMC11781716; doi:10.1371/journal.pone.0315592)
Supplement: Attachment — Submitted filename: PONE-D-24-03861 (R-FHH)-06 June 2024.pdf [file pone.0315592.s004.pdf]

PLOS ONE

The involvement of nongovernmental organisations in achieving health system goals based on the WHO six building blocks: A scoping review

--Manuscript Draft--

**Manuscript Number:**	PONE-D-24-03861
**Article Type:**	Research Article
**Full Title:**	The involvement of nongovernmental organisations in achieving health system goals based on the WHO six building blocks: A scoping review
**Short Title:**	The involvement of nongovernmental organisations in achieving health system goals
**Corresponding Author:**	Edris Kakemam<br>Tabriz University of Medical Sciences<br>Tabriz, East Azerbaijan IRAN, ISLAMIC REPUBLIC OF
**Keywords:**	Non-governmental organization; NGOs; health system; building blocks
**Abstract:**	Background<br><br>Non-governmental organisations (NGOs) have the potential to make a significant contribution to improving health system goals through the provision of resources, health services and community participation. Therefore, this paper examines the role of NGOs in achieving health system goals, based on the six building blocks of a health system framework, and identifies strategies to enhance NGO involvement in achieving health system goals.<br><br>Methods<br><br>A scoping systematic review methodology was used to map and synthesise the existing literature on the topic, following the latest JBI six-stage framework. Five databases and search engines including PubMed, Web of Science (ISI), EMBASE, Scopus and Google Scholar were searched from January 2000 to December 2023. The results were synthesised using a directed content analysis approach, and the findings were categorised according to the dimensions of the six building blocks.<br><br>Results<br><br>NGO involvement in health system goals can effectively address gaps in service delivery, strengthen the health workforce, improve health information systems, increase access to essential medicines, mobilise resources and promote good governance. In addition, six key strategies were identified, including joint planning, policy development, capacity building, resource allocation, developing collaboration, and improving the quality of health care, to enhance NGO participation in achieving health system goals.<br><br>Conclusion<br><br>NGOs can play a critical role in achieving health system goals, alongside government and other key health stakeholders. It is important for governments to use evidence-based policies and interventions to support NGOs to realise their potential in achieving health system goals.
**Order of Authors:**	Leila Doshmangir
	Arman Sanadghol
	Edris Kakemam
	Reza Majdzadeh
**Additional Information:**	
**Question**	**Response**

Powered by Editorial Manager® and ProduXion Manager® from Aries Systems Corporation

**Financial Disclosure**<br><br>Enter a financial disclosure statement that describes the sources of funding for the work included in this submission. Review the submission guidelines for detailed requirements. View published research articles from *PLOS ONE* for specific examples.<br><br>This statement is required for submission and **will appear in the published article** if the submission is accepted. Please make sure it is accurate.<br><br>**Funded studies**<br>Enter a statement with the following details:<br>• Initials of the authors who received each award<br>• Grant numbers awarded to each author<br>• The full name of each funder<br>• URL of each funder website<br>• Did the sponsors or funders play any role in the study design, data collection and analysis, decision to publish, or preparation of the manuscript?<br><br>Did you receive funding for this work?	The author(s) received no specific funding for this work.
**Competing Interests**<br><br>Use the instructions below to enter a competing interest statement for this submission. On behalf of all authors, disclose any competing interests that could be perceived to bias this work—acknowledging all financial support and any other relevant financial or non-financial competing interests.<br><br>This statement is required for submission and **will appear in the published article** if the submission is accepted. Please make sure it is accurate and that any funding sources listed in your Funding Information later in the submission form are also declared in your Financial Disclosure statement.	The authors have declared that no competing interests exist.

View published research articles from *PLOS ONE* for specific examples.<br><br>**NO authors have competing interests**<br><br>Enter: *The authors have declared that no competing interests exist.*<br><br>**Authors with competing interests**<br><br>Enter competing interest details beginning with this statement:<br><br>*I have read the journal's policy and the authors of this manuscript have the following competing interests: [insert competing interests here]*<br><br>* typeset	
**Ethics Statement**<br><br>Enter an ethics statement for this submission. This statement is required if the study involved:<br><br>• Human participants<br>• Human specimens or tissue<br>• Vertebrate animals or cephalopods<br>• Vertebrate embryos or tissues<br>• Field research<br><br>Write "N/A" if the submission does not require an ethics statement.<br><br>General guidance is provided below. Consult the submission guidelines for detailed instructions. **Make sure that all information entered here is included in the Methods section of the manuscript.**	N/A

**Format for specific study types**<br><br>**Human Subject Research (involving human participants and/or tissue)**<br>• Give the name of the institutional review board or ethics committee that approved the study<br>• Include the approval number and/or a statement indicating approval of this research<br>• Indicate the form of consent obtained (written/oral) or the reason that consent was not obtained (e.g. the data were analyzed anonymously)<br><br>**Animal Research (involving vertebrate animals, embryos or tissues)**<br>• Provide the name of the Institutional Animal Care and Use Committee (IACUC) or other relevant ethics board that reviewed the study protocol, and indicate whether they approved this research or granted a formal waiver of ethical approval<br>• Include an approval number if one was obtained<br>• If the study involved *non-human primates*, add *additional details* about animal welfare and steps taken to ameliorate suffering<br>• If anesthesia, euthanasia, or any kind of animal sacrifice is part of the study, include briefly which substances and/or methods were applied<br><br>**Field Research**<br><br>Include the following details if this study involves the collection of plant, animal, or other materials from a natural setting:<br>• Field permit number<br>• Name of the institution or relevant body that granted permission	
**Data Availability**<br><br>Authors are required to make all data underlying the findings described fully available, without restriction, and from the time of publication. PLOS allows rare exceptions to address legal and ethical concerns. See the PLOS Data Policy and FAQ for detailed information.	Yes - all data are fully available without restriction

A Data Availability Statement describing where the data can be found is required at submission. Your answers to this question constitute the Data Availability Statement and **will be published in the article**, if accepted.<br><br>**Important:** Stating 'data available on request from the author' is not sufficient. If your data are only available upon request, select 'No' for the first question and explain your exceptional situation in the text box.<br><br>Do the authors confirm that all data underlying the findings described in their manuscript are fully available without restriction?	
**Describe where the data may be found in full sentences. If you are copying our sample text, replace any instances of XXX with the appropriate details.**<br><br>• If the data are **held or will be held in a public repository**, include URLs, accession numbers or DOIs. If this information will only be available after acceptance, indicate this by ticking the box below. For example: *All XXX files are available from the XXX database (accession number(s) XXX, XXX.)*.<br>• If the data are all contained **within the manuscript and/or Supporting Information files**, enter the following: *All relevant data are within the manuscript and its Supporting Information files.*<br>• If neither of these applies but you are able to provide **details of access elsewhere**, with or without limitations, please do so. For example:<br><br>*Data cannot be shared publicly because of [XXX]. Data are available from the XXX Institutional Data Access / Ethics Committee (contact via XXX) for researchers who meet the criteria for access to confidential data.*<br><br>*The data underlying the results presented in the study are available from (include the name of the third party*	All relevant data are within the manuscript and its Supporting Information files.

and contact information or URL).<br>• This text is appropriate if the data are owned by a third party and authors do not have permission to share the data.<br><br>* typeset	
Additional data availability information:	

Manuscript Click here to access/download;Manuscript;Manuscript.docx

The involvement of nongovernmental organisations in achieving health system goals based on the WHO six building blocks: A scoping review

Abstract

Background: Non-governmental organisations (NGOs) have the potential to make a significant contribution to improving health system goals through the provision of resources, health services and community participation. Therefore, this paper examines the role of NGOs in achieving health system goals, based on the six building blocks of a health system framework, and identifies strategies to enhance NGO involvement in achieving health system goals.

Methods: A scoping systematic review methodology was used to map and synthesise the existing literature on the topic, following the latest JBI six-stage framework. Five databases and search engines including PubMed, Web of Science (ISI), EMBASE, Scopus and Google Scholar were searched from January 2000 to December 2023. The results were synthesised using a directed content analysis approach, and the findings were categorised according to the dimensions of the six building blocks.

Results: NGO involvement in health system goals can effectively address gaps in service delivery, strengthen the health workforce, improve health information systems, increase access to essential medicines, mobilise resources and promote good governance. In addition, six key strategies were identified, including joint planning, policy development, capacity building, resource allocation, developing collaboration, and improving the quality of health care, to enhance NGO participation in achieving health system goals.

Conclusion: NGOs can play a critical role in achieving health system goals, alongside government and other key health stakeholders. It is important for governments to use evidence-based policies and interventions to support NGOs to realise their potential in achieving health system goals.

Keywords: Non-governmental organization, NGOs, health system, building blocks

Introduction

Non-governmental organizations (NGOs) play a vital role in the achievement of health system goals [1] and ensuring that everyone has access to healthcare [2]. Through their contributions to health system financing, they help bridge the financial gaps[3], reduce inequalities [4], and enhance the overall accessibility [5] and quality of healthcare services [6]. In other words, NGOs are essential in achieving health system goals by addressing gaps, collaborating with government entities, and contributing to the enhancement of various components of healthcare systems [7]. Their efforts in delivering services [8], health workforce [9], health information systems [10], medical products, vaccines, and technologies [11], as well as contributing to health system financing[12], have a significant impact on the overall effectiveness and accessibility of healthcare [13]. Recognizing and supporting the invaluable role of NGOs is crucial for establishing sustainable and inclusive health systems that can effectively meet the needs of communities [14]. By working together, NGOs and governments can build stronger healthcare systems and ensure better health outcomes for all [15]. NGOs play a crucial role in addressing healthcare challenges [16], and since healthcare systems strive to provide equitable [17], accessible [18], and quality care to populations [19], NGOs serve as essential partners in complementing and supporting government, other stakeholders [20], other stakeholders [21], and protecting the rights of marginalized populations [22]. Therefore, their efforts not only complement the work of governments but also offer innovative solutions to complex challenges [23].

NGOs play a vital role in the financing of the health system by raising funds from various sources [24], including donors, philanthropists, and corporate partnerships [25]. They bridge the financial gaps that governments may not be fully able to cover [26], ensuring that everyone, regardless of their socio-economic status, can access essential healthcare services [27]. Additionally, NGOs actively strive to reduce disparities in healthcare[28] by targeting underserved communities and marginalized populations [29]. They implement programs and initiatives that specifically cater to the unique needs of these groups [30], guaranteeing that they have fair access to healthcare services [31]. Apart from addressing financial gaps and reducing disparities, NGOs also contribute to enhancing the overall accessibility of healthcare services [32]. They establish clinics within

communities [33], deploy mobile health units [34], and implement telemedicine programs in remote areas where traditional healthcare infrastructure is lacking [35].

NGOs are autonomous organizations that function without government regulation [36]. They are frequently motivated by a specific mission or cause and strive to enhance society through various means [37]. In the realm of healthcare, NGOs can concentrate on a wide array of issues, ranging from facilitating access to healthcare services to conducting research and engaging in advocacy [38]. NGOs bring a distinct perspective and expertise to the table when it comes to healthcare [39]. They are often intimately connected to the communities they serve and possess a profound comprehension of the local circumstances and requirements [40]. This grants them the flexibility and capacity to innovate [41], unlike governmental entities, enabling them to promptly address emerging health challenges and adapt their strategies accordingly [42]. Additionally, NGOs often act as champions for marginalized populations, ensuring that their voices are acknowledged and their needs are met within the healthcare system [43].

The World Health Organization (WHO) has identified six building blocks that serve specific purposes and contribute to the overall functioning of the health system [44]. Each of these building blocks plays a crucial role in achieving the goals of the health system, and NGOs can actively contribute to each of them. In this scoping review, we explore the role of NGOs in achieving health system goals based on the six building blocks of a health system framework and strategies to enhance the participation of NGOs in achieving these goals. By examining each building block - service delivery, health workforce, health information systems, medical products, vaccines, and technologies, and health system financing - we delve into the significant contributions that NGOs make in improving healthcare outcomes and expanding access to essential services. Understanding the importance of NGOs in healthcare is essential for effective collaboration and informed decision-making to ensure the overall strengthening of health systems worldwide.

Method

Since our goal is to analyze the literature on the role of NGOs in achieving health system goals using the six building blocks of a health system framework, we utilized the latest JBI guidance for scoping reviews to conduct this review [45].

This approach allowed us to effectively summarize the findings and identify gaps in existing research. The framework comprises six sequential stages: (1) Formulating the research question, (2) identifying relevant studies, (3) setting inclusion criteria, (4) screening and selecting evidence, (5) extracting data, and (6) analyzing data. To present the results, we followed the PRISMA-ScR (Preferred Reporting Items for Systematic reviews and Meta-Analyses extension for Scoping Reviews) checklist (refer to Appendix S1 for more details) [46].

Step one: Identifying the research question

A scoping review typically begins with one or more questions. Therefore, the current scoping review aimed to address the following inquiries:

1-What is the role of non-governmental organizations (NGOs) in accomplishing health system goals in different contexts? Specifically, our focus was on the role of NGOs in the health system, specifically in relation to the six building blocks: service delivery, health workforce, health information systems, medical products, vaccines, and technologies, and health system financing.

2-What strategies do NGOs employ to engage and achieve their objectives?

Step 2. Identifying relevant studies

Four databases including PubMed, Web of Science (ISI), EMBASE, Scopus, and Google Scholar search engine were searched from January 2000 to December 2023. The research team identified keywords from the studies and then collaborated with a librarian to develop a search strategy. In January 2024, a literature search was conducted using the terms "non-governmental organization," "non-state provider," "non-government sector," "health service," "health system," and "health service delivery" in the PubMed, Web of Science (ISI), EMBASE, and Scopus databases, and Google Scholar search engine. The keywords in the peer-reviewed literature search were combined using the Boolean terms "AND" and "OR" in all the explored electronic databases. Supplementary file 1 contains a comprehensive list of the searches carried out. To ensure comprehensiveness in the literature reviews, we manually searched for references within the selected articles.

Step 3. Inclusion criteria

Full articles were considered eligible if they met the following criteria: (i) they were published in English, (ii) they were conducted between 2000 and December 31, 2023, (iii) they were published in peer-reviewed journals, (iv) they were either original research or review studies, and (v) they included sufficient data to analyze the impactful interventions of NGOs in health-related activities and the health system. On the other hand, studies were excluded if they (i) were not available as full-text, (ii) were not peer-reviewed and were only published as abstracts, conference proceedings, summaries, letters to the editor, commentaries, or opinion pieces, and (iii) lacked quantitative or qualitative details. The screening and selection of studies were carried out independently by two authors, based on the inclusion and exclusion criteria.

Step 4. Evidence screening and selection

The results obtained from the bibliographic databases and hand searching were merged, and any duplicate entries were eliminated. LD and EK independently reviewed the search results based on the title, abstract, and full text. In cases where there were disagreements between the reviewers during the paper review and selection process, a third reviewer (EK) was involved to make the final decision. Following scoping review methodology, we did not conduct any quality assessment or risk of bias assessment for the included studies, as our aim is to quickly map the evidence. [45].

Step 5. Data extraction

We created a data extraction form. We extracted and organised details of studies such as first author, country, date, type, study design, quality assessment, field of activity, intervention(s), implementation considerations, role of NGOs based on the six building blocks and strategies using Microsoft Excel. Two reviewers (LD and EK) extracted relevant data from the retrieved articles for a narrative synthesis. In case of disagreement between the reviewers during the data extraction process, a third reviewer (RM) was involved to make the final decision.

Step 6. Data analysis

We conducted a directed content analysis to synthesise the results and categorise the findings according to the dimensions of the six building blocks. The identified items were categorised into sub-themes using deductive reasoning. To establish the primary categorisation of the recommended codes, we drew on existing research on NGO involvement in the health system.

Result

Selection of evidence sources

The process of reviewing and presenting the search results is shown in Figure 1. Initially, a keyword search yielded 1424 articles. After screening the abstracts, 112 studies were selected for a thorough review of the full text. Data extraction and analysis was then performed on 86 studies published between 2000 and 2023.

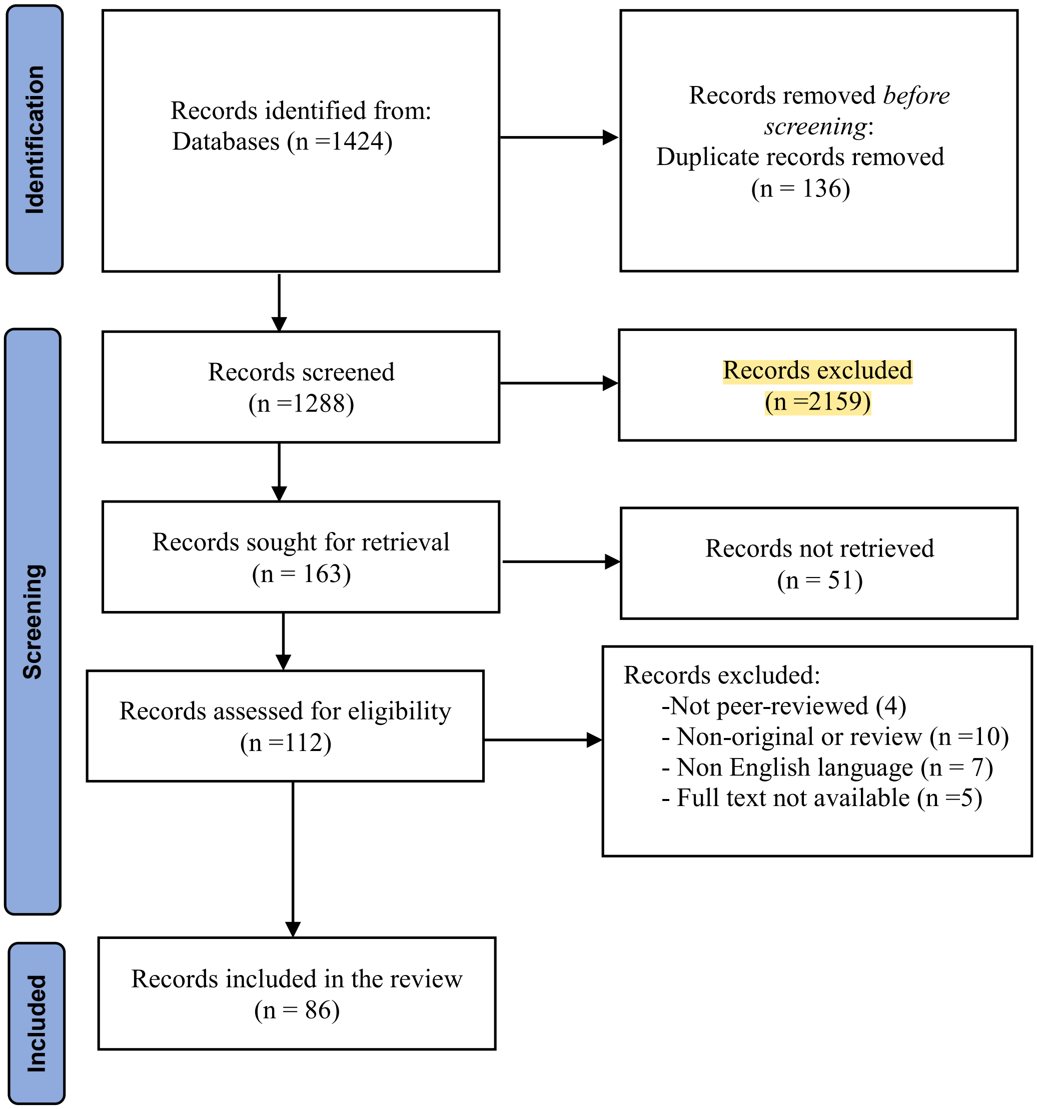

Characteristics of evidence sources

Studies were conducted in 51 countries and five regions (Eastern and Central Europe, Sub-Saharan Africa, South and Southeast Asia, the Caribbean and Latin America). Ten studies were conducted in high-income countries, 20 in upper-middle-income countries, 51 in LMICs and 20 in low-income countries. 85 studies reported that NGOs provided primary care, 4 studies referred to secondary care, and 8 studies referred to tertiary care. Based on the Health Research Classification System (HRCS)[47], there were studies on 8 of the 21 HRCS categories. 32 studies were on general health topics, 25 studies were on infectious diseases, 22 studies were on reproductive health and childbirth, 4 studies were on mental health topics, 3 studies were on cancer and neoplasms, 2 studies were on cardiovascular topics, 3 studies were on inflammation and the immune system, and 1 study was on musculoskeletal topics (more details in Supplementary file 2).

Synthesis of results

On the basis of the six building blocks, eighteen cases of intervention were identified related to the involvement of NGOs in achieving health system objectives. (Table 2). Also, 6 themes, 16 sub-themes and 45 strategies of NGO participation in achieving health system goals were identified. (Table 2).

Table 2. NGOs participation base on the Six Building Blocks

**Main blocks**		**Subscales**	**Number of studies**
Service delivery		Health care	29
		Health facilities	33
		Special diseases	69
Health workforce		Health care professionals	37
		Community Health Worker/ Community	44
Information		Health- oriented programs	35
		Information support	1
Medical products, vaccines and technologies		Medical products	24
		Vaccines	7
		Equipment and technologies	6
Financing	Financing source	Free/ subside	33
		Insurance	7
	Financial support	Donation/ Grant	20
		International organization	15
		Self-Financing	8
Leadership and governance		Interact with State	31
		Interact with health service providers	17
		Interact with citizen	10

Service delivery

One of the factors influencing NGO involvement in achieving health system goals is service delivery. NGOs provided services through the provision of health facilities, health care services and services for specific conditions. 85 studies focused on NGO service delivery [48-125], of which 29 studies focused on health care services, 69 studies focused on providing services for specific diseases (such as HIV/AIDS, Ebola, tuberculosis, reproductive, cardiovascular), and 33 studies focused on providing health facilities as strategies for NGO involvement.

Health workforce

Through a variety of strategies and interventions, NGOs provide or support the provision of health services or the operation of health facilities by providing skilled personnel. Fifty-eight studies focused on health workers through NGO involvement [48, 50-52, 54-57, 59, 61, 63-68, 74-77, 79, 81-85, 89-98, 101-105, 111-113, 117, 119-123, 125-130]. Of these, 37 refer to the use of health professionals and 44 studies refer specifically to community health workers/communities.

Health information systems

NGOs use different sources of information needed to promote public health and raise awareness among the target population. Thirty-three studies focused on the third core component of the building blocks, health information systems [50, 53, 63, 66, 72, 74, 79, 81, 82, 84, 87, 88, 90, 93, 95-98, 101-105, 108, 109, 117, 118, 122, 126-129, 131]. Of these, all 35 studies reported that NGOs provided health-oriented programmes through awareness raising, training, mobile phone use, outreach, counselling and the like, and only one study reported that NGOs provided information.

Medical products, vaccines and technologies

In many countries, poor people still have limited access to primary health care. NGOs have developed strategies and interventions to improve people's access to health services, especially for the vulnerable, the poor and those with specific diseases. Interventions using medical supplies allow them to treat a wide range of health problems, reducing mortality and morbidity. 27 studies

reported that NGOs use medical products, vaccines, and technologies [48-51, 53, 56, 65, 68, 74, 76, 77, 79, 82, 84, 86, 88, 92, 94, 98, 101-104, 107, 108, 117, 120]. Of these studies, about 24 reported that NGOs provided medical products and seven reported that NGOs provided vaccines. Six studies focused on NGOs providing technology to their target population.

Financing

Protecting people from financial hardship has been achieved by supporting them through a range of strategies, including the provision of free health services, the introduction of insurance schemes or the provision of subsidies. The fifth of the six building blocks of the WHO framework, financing, was the focus of 43 studies [48-53, 55, 56, 58-60, 63, 64, 66, 67, 69, 70, 73-76, 79-85, 88, 92, 97, 99, 100, 102-104, 111, 117, 119, 120, 123, 132]. The component was divided into two parts, 36 studies focusing on the financial support provided by NGOs to their target population and 27 studies focusing on the sources of NGO funding. Of these, some 32 studies reported that NGOs provided free or subsidised services and seven studies reported that NGOs provided insurance plans. 20 studies reported that NGOs received donations or grants, 15 studies reported that NGOs received funding from international organisations and 8 studies reported that NGOs were self-financing in various ways.

Leadership and governance

NGOs build coalitions at different levels, they are at the state level and in contact with health policy makers, with health service providers, and also to be the voice of the people, to be in contact with the community. 42 studies referred to the role of NGOs in leadership and governance [49-51, 53-57, 60, 61, 63, 66, 69, 72, 77, 78, 81, 88, 90, 91, 95-98, 101-104, 109, 112, 114, 118-124, 126, 129, 130, 132].Of these, 31 studies focused on the level of NGO interaction at the government level, 17 studies at the level of health care providers, and 10 studies at the level of citizens.

Strategies for involving NGOs in achieving health system goals

The strategies identified through the review for improving NGO participation in achieving the goals of the health system included 6 themes: joint planning, policy development, capacity building, resource allocation, developing cooperation, and improving the quality of health care. Some 45 strategies were also identified, grouped into 16 sub-themes. Further details are given in Table 3.

Table 3. Themes, sub-themes, and strategies of NGOs' participation in achieving the goals of the health system

Theme	Sub-theme	Strategies
**Joint planning**	**Increase access**	-Providing health services/promotion for community [48-58, 63-77, 79-100, 105, 107, 110-113, 115-118, 120-125, 129, 131, 132]<br>-Providing health facilities [56, 63, 77, 81, 122]
	**Increase equity**	-Strengthening the health system governance [63, 81, 104]<br>-Providing equitable cares [78, 94, 103, 122]
**Policy development**	**Aligning objectives**	-Focusing on specific goals [78, 102]
	**Supporting NGOs**	-Advocating for the role of NGOs [60, 102, 128]<br>-Accompanying NGOs by government agencies [104]<br>-Identification of priorities by the MOH and outsourcing them to NGOs [102, 106]<br>-Distribution of prevention materials [68]<br>-Overcoming the reluctance of policy makers and health researchers to acknowledge the abuses and failures of the current model of NGO cooperation [106]<br>-Supporting local NGOs [60, 126]
	**Preparing policy tools**	-Defining roles and responsibilities [62, 97, 102, 122]<br>-Defining nature and schedule of delivery from NGOs for section health [97]<br>-Defining a plan for scaling and sustainable development for NGOs from government [97, 102]<br>-Ensuring political support and policymakers' trust in NGOs [78]
	**Community-based interventions**	-Conducting interventions through community participation [98, 111, 112, 124]<br>-Conducting interventions through CHWs [63, 90, 105, 125]
**Capacity building**	**Training and education healthcare workers**	-Considering financial and nonfinancial incentives, especially tangible rewards [78, 90]<br>-Providing clinical education [84]<br>- Training provision for CHWs [90, 105, 127]
	**Raising awareness of the community**	- Community health education [53, 68, 72, 79, 87, 94, 118]<br>- Community awareness and training [79, 81, 87, 93, 118]<br>-Editing and preparation of prevention information materials [68]<br>- Distribution of information materials [68]
	**Empowering NGOs**	-Organising training courses by NGOs to improve knowledge and skills [68]<br>-Implementing creative and culturally appropriate preventive interventions [62]

**Resource allocation**	**Promoting innovative models and mechanisms**	- Lending to the poor [81]<br>- Using mobile phones to communicate with patients [82]<br>- Providing nursing homes for patients [110, 118]
	**Expand financial coverage**	- Funding through community-based health insurance programmes for the community [66, 67]<br>-Financial support for the community [81, 93, 107, 131]
	**Sharing resources**	-Sharing of information between stakeholders [114]<br>-Commitment to mobilise resources [114]<br>-Sharing physical resources [71, 81, 84, 121]
**Collaboration development**	**Promotion participation**	- Developing cooperation between government agencies, NGOs and the private sector [78, 81, 108, 119]<br>-Strengthening intersectoral coordination [81, 102, 104, 122]<br>-Establishing transparent and accountable structures and mechanisms for coordination [97, 102, 104, 106]<br>-Investing in a long-term relationship [102, 104, 106, 114]<br>-Building new relationships by adopting and implementing evidence-based health interventions [114]<br>- Using decentralised structures to promote local ownership and sustainability of programmes [114]
	**Developing communication channels**	- Strengthening networking relationships between NGOs [81]<br>- Promoting links between NGOs [68, 104]
**Improvement quality healthcare**	**Supporting quality assurance measures**	-Establish a mechanism to assess the quality of health care [102, 126]<br>-Establish and strengthen effective monitoring and evaluation of the quality of health services[114]

Discussion

We systematically reviewed studies that examined the role of NGOs and their engagement strategies in achieving health system goals based on the six building blocks of the WHO framework. We found that, NGOs are often at the forefront of addressing health system gaps and challenges within the health system [78]. They have the flexibility to identify areas for improvement and implement innovative solutions [133]. For example, in regions with limited access to health services, NGOs can set up mobile clinics or community health centres to reach underserved populations [134]. NGOs can also focus on specific health issues, such as maternal and child health or infectious diseases, and design targeted interventions to address these challenges [135]. In this context, we found that most NGOs aim to expand service delivery, improve financing and provide essential medical products to help meet the health needs of countries' populations.[103].

Collaboration between NGOs and government agencies is key to achieving health system goals. While NGOs bring expertise and grassroots understanding, governments have the resources and power to implement systemic change [109]. By working together, NGOs and government agencies can leverage their respective strengths and create a more comprehensive and effective health system [114]. This collaboration can include joint planning, sharing of resources and coordination of efforts to maximise impact and ensure that health system goals are achieved [120]. This finding is consistent with the policy framework on multi-stakeholder partnerships, which emphasizes the importance of inclusive decision-making, joint planning and shared resources to achieve sustainable development [136]. On the other hand, the public-private partnership (PPP) framework allows government to involve NGOs On the other hand, the public-private partnership (PPP) framework allows government to involve NGOs [137]. This framework uses the resources and skills of NGOs, governments and the private sector to achieve common goals, such as the development of health infrastructure and the provision of social health services [138]. Of course, it's important to note that these frameworks can vary according to the specific political, social and cultural contexts within a country or region [139].

Over the years, significant reforms have been undertaken with regard to NGOs in the health sector [140]. These reforms aim to strengthen the capacity and effectiveness of NGOs in health service delivery, ensure transparency and accountability, and promote collaboration with government and

other stakeholders [101]. One of these reforms is strengthening the capacity of NGOs; governments have recognised the importance of increasing the competence of NGOs to manage health services effectively [104]. Capacity-building efforts focus on increasing the skills, supervision, technical expertise and knowledge of NGO staff [141]. Initiatives such as training programmes, seminars and consultancy services are being expanded to improve their ability to design, implement and evaluate health interventions [142]. NGOs have provided competent personnel for the delivery of health services, using a mix of external and internal stimuli; for example, it has been shown that the act of decision-making, the organisation's vision, mission and strategy, and the competencies and skills of NGO personnel have a positive impact on the effectiveness of NGOs in the delivery of health services [59, 120].

On the other hand, reforms related to NGOs in the health system have been implemented by various countries worldwide [101]. These reforms aim to improve the overall effectiveness, transparency and accountability of NGOs in the delivery of health services [104]. For example, India has one of the largest NGO sectors in the world and plays a significant role in the delivery of health services, particularly in rural and marginalised communities [132]. To address the challenges of unregulated NGOs, the Indian government launched the National Health Mission (NHM) in 2013 [143].

The NHM aims to strengthen the health system, including through better engagement of NGOs [143]. Reforms introduced under the NHM include NGO accreditation, results-based funding, and regular monitoring and evaluation of NGO activities [144]. In Kenya, health system reforms have been implemented to increase the effectiveness and streamline the work of NGOs [145]. Key reforms include the creation of a legal framework to regulate NGO activities, the establishment of a partnership and coordination framework with NGOs, and performance-based funding [146]. In Australia, the government has introduced a competitive grant system whereby NGOs must demonstrate their ability to deliver effective and evidence-based health services in order to receive funding. This ensures rigorous scrutiny and quality control before funds are allocated [135]. These examples show how different countries have introduced reforms to strengthen the role and effectiveness of NGOs in the health system. These reforms increase accountability, promote coordination and ensure better health outcomes for the target populations.

Governments have established mechanisms to provide financial support to NGOs working in the health sector [61]. These include funding opportunities through grants, contracts and subsidies [141]. Financial reforms aim to improve the availability of resources to NGOs, enabling them to deliver health services effectively, invest in infrastructure and recruit skilled staff [102]. One of the mechanisms used by NGOs in collaboration with government in recent years is social health insurance (SHI). Social health insurance schemes are typically structured so that individuals make financial contributions to a centralised fund [147, 148]. These contributions can be made either indirectly, through taxes, or directly, through wage-based payments [149]. However, because of the financial constraints faced by certain groups of the population, many countries have introduced hybrid SHI systems [150, 151].

NGOs often use health information to tailor their programmes, allocate resources effectively and respond to the specific needs of communities [152]. In Ghana, for example, NGOs such as the Ghana Health Service and the Navrongo Health Research Centre rely heavily on health information to improve health services. These organisations collect data on disease prevalence, mortality and health infrastructure to identify priority areas [153]. In India, NGOs such as Pratham and PATH have used health information to address specific issues [154].). Health data helps NGOs in India identify prevalent diseases such as tuberculosis, malnutrition or cataracts and design appropriate interventions [154]. In both Ghana and India, NGOs work with local governments and international health organisations to collect and analyse data to ensure that their programmes are aligned with national health policies and goals. However, there are potential challenges to the use of health information by NGOs [101]. Some NGOs may have difficulty accessing reliable and up-to-date data in resource-limited settings [114]. There may also be concerns about data privacy, security and sharing between NGOs and government agencies [155].

The use of health governance and leadership by NGOs is crucial in addressing health challenges in many countries [156]. Through advocacy, service delivery and capacity building, these organisations contribute to improving health systems and health outcomes [157]. Examples from countries such as India and Kenya illustrate the important role of NGOs in health governance and leadership. In India, NGOs such as the Indian Health Organization (IHO) work with the government to address a range of health challenges, including maternal and child health, HIV prevention and rural health services [158]. Through their strong governance structures and

leadership, these NGOs play a key role in advocating for policy changes and implementing innovative strategies to improve health outcomes [158]. In Kenya, NGOs such as the Kenya Red Cross Society and AMREF Health Africa actively participate in health governance by engaging with policy-makers, advocating for policy change and mobilising resources for health initiatives [159, 160]. These organisations also provide leadership by implementing community-based health solutions and training health workers, thereby strengthening the health system at the grassroots level [159, 160]. It is important to note, however, that the effectiveness of health governance and leadership by NGOs depends on several factors, including the availability of funding, government support and community commitment [161].

Limitations

Due to the breadth of the topic, our study was limited to published papers and excluded grey literature, which could potentially limit the scope of our review. Nevertheless, we took into account the quality of published studies and included all types of studies (i.e. qualitative, quantitative and mixed methods), which allowed for a comprehensive overview.

Conclusion

This analysis has highlighted the ability of NGOs to play a critical role in achieving health system goals, in collaboration with government and other key health sector actors. These organisations have been actively involved in service delivery, health workforce development, information management, procurement of medical products, vaccines and technologies, and leadership and governance. NGOs and governments can work together to build partnerships, establish effective channels of communication and align their goals. This collaboration can include joint planning, policy development, capacity building and resource allocation to achieve common health system goals and ensure equitable access to health services. By working together, NGOs and governments can leverage their strengths, pool resources and coordinate efforts to build more comprehensive and sustainable health systems. The involvement of NGOs in the pursuit of health system goals based on the six essential building blocks is essential. Their involvement can address gaps in service delivery, strengthen the health workforce, improve health information systems, facilitate access to essential medicines, mobilise resources and promote good governance. Governments can benefit from the expertise and resources of NGOs by partnering with them to achieve better health outcomes for all.

References

1. Perry HB, Chowdhury M, Were M, LeBan K, Crigler L, Lewin S, et al. Community health workers at the dawn of a new era: 11. CHWs leading the way to "Health for All". Health Research Policy and Systems 2021;19:1-21.

2. Hémono R, Relyea B, Scott J, Khaddaj S, Douka A, Wringe A. "The needs have clearly evolved as time has gone on.": A qualitative study to explore stakeholders' perspectives on the health needs of Syrian refugees in Greece following the 2016 European Union-Turkey agreement. Conflict and Health 2018;12(1):1-9.

3. Quaye I, Abrokwah E, Sarbah A, Osei JY. Bridging the SME financing gap in Ghana: The role of microfinance institutions. Open Journal of Business and Management 2014;2(04):339.

4. Fezeu F, Awad AJ, Przybylowski CJ, Starke RM, Grober Y, Schlesinger D, et al. Access to stereotactic radiosurgery: identification of existing disparities and a modest proposal to reduce them. Cureus 2014;6.(1): e157.

5. Heiss A, Kelley J. Between a rock and a hard place: International NGOs and the dual pressures of donors and host governments. The Journal of Politics 2017;79(2):732-41.

6. Veenema TG, Rains AB, Casey-Lockyer M, Springer J, Kowal M. Quality of healthcare services provided in disaster shelters: An integrative literature review. International emergency nursing 2015;23(3):225-31.

7. Sacks E, Morrow M, Story WT, Shelley KD, Shanklin D, Rahimtoola M, et al. Beyond the building blocks: integrating community roles into health systems frameworks to achieve health for all. BMJ global health 2018;3(Suppl3): e001384.

.8 Chowdhury TA, Das AB, Chakraborty L, Barai MK. NGOs for development: experience of Bangladesh. Bangladesh's Economic and Social Progress: From a Basket Case to a Development Model 2020:351-80.

9. Najjar S, Hafez S, Al Basuoni A, Obaid HA, Mughnnamin I, Falana H, et al. Stakeholders' Perception of the Palestinian Health Workforce Accreditation and Regulation System: A Focus on Conceptualization, Influencing Factors and Barriers, and the Way Forward. International journal of environmental research and public health 2022;19(13):8131.

10. Gebre-Mariam M, Bygstad B. Digitalization mechanisms of health management information systems in developing countries. Information and Organization 2019;29(1):1-22.

11. World Health Organization. WHO essential medicines and health products: annual report 2015. World Health Organization; 2016. https://iris.who.int/bitstream/handle/10665/252838/WHO-EMP-2016.02-eng.pdf

12. Whyle EB, Olivier J. Models of public–private engagement for health services delivery and financing in Southern Africa: a systematic review. Health policy and planning 2016;31.29-1515:(10)

13. Atar E, Hossain F, Ullah AA. Syrian refugees in Turkey: exploring the role of I/NGOs in refugee crisis. Third World Quarterly 2023;44(2):231-45.

14. World Health Organization. Health policy and system support to optimize community health worker programmes for HIV, TB and malaria services: an evidence guide. 2020. https://iris.who.int/bitstream/handle/10665/340078/9789240018082-eng.pdf?sequence=1
15. Masefield SC, Msosa A, Grugel J. Challenges to effective governance in a low income healthcare system: a qualitative study of stakeholder perceptions in Malawi. BMC health services research 2020;20(1):1-16.
16. Chiarenza A, Dauvrin M, Chiesa V, Baatout S, Verrept H. Supporting access to healthcare for refugees and migrants in European countries under particular migratory pressure. BMC health services research 2019;19(1):1-14.
17. Jensen N, Kelly A, Avendano M. Health equity and health system strengthening–Time for a WHO re-think. Global Public Health 2022;17(3):377-90.
18. Rudebeck CE. Relationship based care–how general practice developed and why it is undermined within contemporary healthcare systems. Scandinavian Journal of Primary Health Care 2019;37(3):335-44.
19. Kruk ME, Gage AD, Joseph NT, Danaei G, García-Saisó S, Salomon JA. Mortality due to low-quality health systems in the universal health coverage era: a systematic analysis of amenable deaths in 137 countries. The Lancet 2018;392(10160):2203-12.
20. Toepler S, Zimmer A, Levy K, Fröhlich C. Beyond the partnership paradigm: Toward an extended typology of government/nonprofit relationship patterns. Nonprofit and Voluntary Sector Quarterly 2023;52(1_suppl):160S-86S.
21. Yan X, Lin H, Clarke A. Cross-sector social partnerships for social change: The roles of non-governmental organizations. Sustainability 2018;10(2):558.
22. Erdilmen M, Sosthenes WA. Opportunities and challenges for localization of humanitarian action in Tanzania. Local Engagement Refugee Research Network Paper 2020;8:1-35.
23. Adem SA, Childerhouse P, Egbelakin T, Wang B. International and local NGO supply chain collaboration: an investigation of the Syrian refugee crises in Jordan. Journal of Humanitarian Logistics and Supply Chain Management 2018;8(3):295-322.
24. Nyasulu J, Pandya H. The effects of coronavirus disease 2019 pandemic on the South African health system: A call to maintain essential health services. African Journal of Primary Health Care and Family Medicine 2020;12(1):1-5.
25. Kumi E. Domestic resource mobilisation strategies of national non□governmental organisations in Ghana. Public Administration and Development 2022;42(2):109-27.
26. Springman J, Malesky E, Right L, Wibbels E. The effect of government repression on civil society: evidence from Cambodia. International Studies Quarterly 2022;66(3):sqac028.
27. Jayasooria D. Sustainable development goals and social work: Opportunities and challenges for social work practice in Malaysia. Journal of Human Rights and Social Work 2016;1(1):19-29.
28. Nanjunda DC. Introspection of Tribal Health Issues and NGOs: Examination of Literatures. Public Affairs And Governance 2016;4(2):135-45.

29. Islam MR. Non-governmental organizations and community development in Bangladesh. International Social Work 2017;60(2):479-93.
30. Brass JN, Longhofer W, Robinson RS, Schnable A. NGOs and international development: A review of thirty-five years of scholarship. World Development 2018;112:136-49.
31. Richard L, Furler J, Densley K, Haggerty J, Russell G, Levesque J-F, Gunn J. Equity of access to primary healthcare for vulnerable populations: the IMPACT international online survey of innovations. International journal for equity in health 2016;15(1):1-20.
32. Doocy S, Lyles E, Akhu-Zaheya L, Burton A, Burnham G. Health service access and utilization among Syrian refugees in Jordan. International journal for equity in health 2016;15(1):1-15.
33. Black RE, Taylor CE, Arole S, Bang A, Bhutta ZA, Chowdhury AMR, et al. Comprehensive review of the evidence regarding the effectiveness of community–based primary health care in improving maternal, neonatal and child health: 8. summary and recommendations of the Expert Panel. Journal of global health 2017;7.(1)
34. Alban A, Blaettchen P, de Vries H, Van Wassenhove LN. Resource allocation with sigmoidal demands: Mobile healthcare units and service adoption. Manufacturing & Service Operations Management 2022;24.61-2944:(6)
35. Yim D, Chandra S, Sondh R, Thottarath S, Sivaprasad S. Barriers in establishing systematic diabetic retinopathy screening through telemedicine in low-and middle-income countries. Indian Journal of Ophthalmology 2021;69(11):2987.
36. Lauwo SG, Otusanya OJ, Bakre O. Corporate social responsibility reporting in the mining sector of Tanzania: (Lack of) government regulatory controls and NGO activism. Accounting, Auditing & Accountability Journal 2016;29(6):1038-74.
37. Arhin A. Advancing post-2015 Sustainable Development Goals in a changing development landscape: Challenges of NGOs in Ghana. Civil Society Sustainability: Routledge; 2018. p. 29-42.
38. Chuah FLH, Tan ST, Yeo J, Legido-Quigley H. Health system responses to the health needs of refugees and asylum-seekers in Malaysia: a qualitative study. International journal of environmental research and public health 2019;16(9):1584.
39. Mercer T, Gardner A, Andama B, Chesoli C, Christoffersen-Deb A, Dick J, et al. Leveraging the power of partnerships: spreading the vision for a population health care delivery model in western Kenya. Globalization and health 2018;14(1):1-11.
40. Nina I, Alla V. Socially oriented NGOs and local communities in a Russian region: ways to build up their relationship. Laboratorium: Журнал социальных исследований 2017(3):82-103.
41. Muhammad KH, Suliman AET, Mazen JAS, Abu-Naser SS. The Reality of Applying Strategic Agility in Palestinian NGOs. International Journal of Academic Multidisciplinary Research 2020.103-76:(4)4;
42. Gillespie AM, Obregon R, El Asawi R, Richey C, Manoncourt E, Joshi K, et al. Social mobilization and community engagement central to the Ebola response in West Africa: lessons for future public health emergencies. Global Health: Science and Practice 2016;4(4):626-46.

43. Schaaf M, Topp SM, Ngulube M. From favours to entitlements: community voice and action and health service quality in Zambia. Health policy and planning 2017;32(6):847-59.
44. World Health Organization. Everybody's business--strengthening health systems to improve health outcomes: WHO's framework for action. 2007. https://iris.who.int/bitstream/handle/10665/43918/9789241596077_eng.pdf
45. Peters MD, Marnie C, Tricco AC, Pollock D, Munn Z, Alexander L, et al. Updated methodological guidance for the conduct of scoping reviews. JBI evidence synthesis 20.26-2119:(10)18;20
46. Tricco AC, Lillie E, Zarin W, O'Brien KK, Colquhoun H, Levac D, et al. PRISMA extension for scoping reviews (PRISMA-ScR): checklist and explanation. Annals of internal medicine 2018;169(7):467-73.
47. UKCRC Health Research Classification System. http://www.hrcsonline.net/. Accessed 10 June 2015.
48. Abdelmoneium AO. Policy and practice: non□governmental organisations and the health delivery system for displaced children in khartoum, Sudan. Child Abuse Review 2010;19(3):203-17.
49. Ahmed N, DeRoeck D, Sadr-Azodi N. Private sector engagement and contributions to immunisation service delivery and coverage in Sudan. BMJ Global Health 2019;4.(2)
50. Albis MLF, Bhadra SK, Chin B. Impact evaluation of contracting primary health care services in urban Bangladesh. BMC Health Services Research 2019;19(1):1-12.
51. Ambrosini M. NGOs and health services for irregular immigrants in Italy: when the protection of human rights challenges the laws. Journal of Immigrant & Refugee Studies 2015;13.34-116:(2)
52. Ament JD, Greene KR, Flores I, Capobianco F, Salas G, Uriona MI, et al. Health impact and economic analysis of NGO-supported neurosurgery in Bolivia. Journal of Neurosurgery: Spine 2014;20(4):436-42.
53. Amirkhanian YA, Kelly J, Benotsch EG, Somlai AM, Brown KD, Fernández MI, Opgenorth KM. HIV prevention nongovernmental organizations in Central and Eastern Europe: programs, resources and challenges. Central European journal of public health 2004;12(1):12-8.
54. Andrade M, Santos T, Andrade L, Oliveira M, Gomes K, Araujo C, et al. Reduction of breast cancer treatment delay as a result of efforts carried out by two Brazilian NGOs. American Society of Clinical Oncology 2018; 4(2).
55. Bader F, Sinha R, Leigh J, Goyal N, Andrews A, Valeeva N, et al. Psychosocial health in displaced Iraqi care-seekers in non-governmental organization clinics in Amman, Jordan: an unmet need. Prehospital and disaster medicine 2009;24(4):312-20.
56. Baig M, Panda B, Das JK, Chauhan AS. Is public private partnership an effective alternative to government in the provision of primary health care? A case study in Odisha. Journal of Health Management 2014;16(1):41-52.

57. Baqui AH, Rosecrans AM, Williams EK, Agrawal PK, Ahmed S, Darmstadt GL, et al. NGO facilitation of a government community-based maternal and neonatal health programme in rural India: improvements in equity. Health policy and planning 2008;23(4):234-43.
58. Barzin Y, editor The role of NGOs in rural Vietnam: a case study and critique. BMC Proceedings 2012; 6(supp 4): 54.
59. Bechange S. Determinants of project success among HIV/AIDS nongovernmental organizations (NGOs) in Rakai, Uganda. The International journal of health planning and management 2010;25(3):215-30.
60. Berenguera A, Pujol-Ribera E, Violan C, Romaguera A, Mansilla R, Giménez A, et al. Core indicators evaluation of effectiveness of HIV-AIDS preventive-control programmes carried out by nongovernmental organizations. A mixed method study. BMC Health Services Research 2011;11.14-1:(1)
61. Biermann O, Eckhardt M, Carlfjord S, Falk M, Forsberg BC. Collaboration between non-governmental organizations and public services in health–a qualitative case study from rural Ecuador. Global Health Action 2016;9(1):32237.
62. Burns PA, Williams MS, Mena LA, Bruce MA, Bender M, Burton ET, Beech BM. Leveraging community engagement: the role of community-based organizations in reducing new HIV infections among Black men who have sex with men. Journal of Racial and Ethnic Health Disparities 2.201-7:193;020
63. Cancedda C, Davis SM, Dierberg KL, Lascher J, Kelly JD, Barrie MB, et al. Strengthening health systems while responding to a health crisis: lessons learned by a nongovernmental organization during the Ebola virus disease epidemic in Sierra Leone. The Journal of infectious diseases 2016;214(suppl_3):S153-S63.
64. De Maio G, Van den Bergh R, Garelli S, Maccagno B, Raddi F, Stefanizzi A, et al. Reaching out to the forgotten: providing access to medical care for the homeless in Italy. International Health 2014;6(2):93-8.
65. De Souza R. Creating "communicative spaces": a case of NGO community organizing for HIV/AIDS prevention. Health communication 2009;24(8):692-702.
66. Devadasan N, Criel B, Van Damme W, Van der Stuyft P, editors. Performance of community health insurance in India: findings from empirical studies. BMC proceedings 2012; 6(1):9.
67. Dhingra R. NGOs and health insurance schemes in India. Health Popul Perspect Issues 2001;24(4):206-17.
68. Ejaz I, Shaikh BT, Rizvi N. NGOs and government partnership for health systems strengthening: a qualitative study presenting viewpoints of government, NGOs and donors in Pakistan. BMC health services research 2011;11(1):1-7.
69. Ferguson J. A non-government organisation and mental health service collaboration for provision of residential comunity-based care.. 2018;52(S1):3–156. Australian & New Zealand Journal of Psychiatry 2018;52(1S):3–156.

70. Fiorini G, Cerri C, Bini S, Rigamonti A, Perlini S, Marazzi N, et al. The burden of chronic noncommunicable diseases in undocumented migrants: A 1-year survey of drugs dispensation by a non–governmental organization in Italy. Public Health 2016;141:26-31.
71. Ghosh SC, Karim F, Ali A, Arif T. The role of NGOs in improving sanitation status in the rural areas of Bangladesh: challenges and expectations. 35th WEDC International Conference, Loughborough 2011:1-8.
72. Gilbert H, Cunliffe A. Non-governmental organisations and the management of HIV and AIDS in refugee camps: A comparison of Marratane camp in Mozambique and Kakuma camp in Kenya. Journal of Contemporary African Studies 2011;29(1):63-81.
73. Gómez-Jauregui J. The feasibility of government partnerships with NGOs in the reproductive health field in Mexico. Reproductive Health Matters 2004;12(24):42-55.
74. Heard A, Awasthi MK ,Ali J, Shukla N, Forsberg BC. Predicting performance in contracting of basic health care to NGOs: experience from large-scale contracting in Uttar Pradesh, India. Health policy and planning 2011;26(suppl_1):i13-i9.
75. Heinmüller R, Dembélé YA, Jouquet G ,Haddad S, Ridde V. Free healthcare provision with an NGO or by the Malian government. Impact on health center attendance by children under five. Field Actions Science Reports The journal of field actions 2013(Special Issue 8).
76. Holland CE, Papworth E, Billong SC, Kassegne S, Petitbon F, Mondoleba V, et al. Access to HIV services at non-governmental and community-based organizations among men who have sex with men (MSM) in Cameroon: An integrated biological and behavioral surveillance analysis. PLoS One 2015;10(4):e0122881.
77. Huff□Rousselle M, Pickering H. Crossing the public–private sector divide with reproductive health in Cambodia: out□patient services in a local NGO and the national MCH clinic. The International Journal of Health Planning and Management 2001;16(1):33-46.
78. Kara H, Arvidson M. To what extent can evaluation frameworks help NGOs to address health inequalities caused by social exclusion? Perspectives in Public Health 2015;135(4):191-6.
79. Kelly JA, Somlai AM, Benotsch EG, Amirkhanian YA, Fernandez MI, Stevenson L, et al. Programmes, resources, and needs of HIV-prevention nongovernmental organizations (NGOs) in Africa, Central/Eastern Europe and Central Asia, Latin America and the Caribbean. AIDS care 2006;18(1):12-21.
80. Khan JA, Ahmed S, MacLennan M, Sarker AR, Sultana M, Rahman H. Benefit incidence analysis of healthcare in Bangladesh–equity matters for universal health coverage. Health policy and planning 2017;32(3):359-65.
81. Khodayari-Zarnaq R, Kakemam E, Arab-Zozani M, Rasouli J, Sokhanvar M. Participation of Iranian non-governmental organizations in health policy-making; barriers and strategies for development. International Journal of Health Governance 2020;25(1):46-56.
82. Mandal N. Telephonic communication in palliative care for better management of terminal cancer patients in rural India: An NGO based approach. Annals of Oncology 2019;30:ix144-ix5.

83. Mehta P, Sharma S, Manglani M. Improvement in pediatric cancer survival in a developing country through collaboration with nongovernmental organization. Indian Journal of Hematology and Blood Transfusion 2013;29(4):331.
84. Mercer A, Khan MH, Daulatuzzaman M, Reid J. Effectiveness of an NGO primary health care programme in rural Bangladesh: evidence from the management information system. Health policy and planning 2004;19(4):187-98.
85. Mercer A, Uddin N, Huq NL, Haseen F, Khan MH, Larson CP. Validating neonatal mortality and use of NGO reproductive health outreach services in rural Bangladesh. Studies in family planning 2006;37(2):111-22.
86. Mercer MA, Thompson SM, de Araujo RM. The role of international NGOs in health systems strengthening: The case of Timor-Leste. International Journal of Health Services 2014;44(2):323-35.
87. Mininim O, Rosemary O. Community based interventions for the reduction of maternal mortality-the role of professional health associations, non-governmental organisations and community-based organisations in delta state, Nigeria. Nigerian Postgraduate Medical Journal 2014;21(4):3.9-43
88. Momoh GT, Oluwasanu MM, Oduola OL, Delano GE, Ladipo OA. Outcome of a reproductive health advocacy mentoring intervention for staff of selected non-governmental organisations in Nigeria. BMC Health Services Research 2015;15:1-9.
89. Mugisha F ,Birungi H, Askew I. Are reproductive health NGOs in Uganda able to engage in the health SWAp? The International Journal of Health Planning and Management 2005;20(3):227-38.
90. Mukherjee JS, Eustache FE. Community health workers as a cornerstone for integrating HIV and primary healthcare. AIDS care 2007;19(sup1):73-82.
91. Nguyen N, Jacobs JP, Dearani JA, Weinstein S, Novick WM, Jacobs ML, et al. Survey of nongovernmental organizations providing pediatric cardiovascular care in low-and middle-income countries. World Journal for Pediatric and Congenital Heart Surgery 2014;5(2):248-55.
92. Nunns D. Working with UK□based non□governmental organisations for better reproductive health in Nepal. BJOG: An International Journal of Obstetrics & Gynaecology 2011; 118:93-5.
93. Odindo MA, Mwanthi MA. Role of governmental and non-governmental organizations in mitigation of stigma and discrimination among HIV/AIDS persons in Kibera, Kenya. East African Journal of Public Health 2008;5(1):1-5.
94. Oleribe OO, Aliyu S ,Taylor-Robinson SD. Is the prevalence of HIV wrongly estimated in Nigeria? Some insights from a 2017 World AIDS day experience from a Nigerian Non-Governmental Organisation. Pan African Medical Journal 2018;29(1):1-5.
95. Perry H, Morrow M, Borger S, Weiss J, DeCoster M, Davis T, Ernst P. Care groups I: an innovative community-based strategy for improving maternal, neonatal, and child health in resource-constrained settings. Global Health: Science and Practice 2015;3(3):358-69.

96. Perry H, Morrow M ,Davis T, Borger S, Weiss J, DeCoster M, et al. Care groups II: a summary of the child survival outcomes achieved using volunteer community health workers in resource-constrained settings. Global Health: Science and Practice 2015;3(3):370-81.
97. Piotrowicz M, Cianciara D. The role of non-governmental organizations in the social and the health system. Przegl Epidemiol 2013;67(1):69-74.
98. Ricca J, Kureshy N, LeBan K, Prosnitz D, Ryan L. Community-based intervention packages facilitated by NGOs demonstrate plausible evidence for child mortality impact. Health Policy and Planning 2014;29(2):204-16.
99. Ridde V, Somé PA, Pirkle CM. NGO-provided free HIV treatment and services in Burkina Faso: scarcity, therapeutic rationality and unfair process. International Journal for Equity in Health 2012;11(1):1-9.
100. Rivera Franco MM, Leon Rodriguez E. Importance of nongovernmental organizations for the establishment of a successful hematopoietic stem-cell transplantation program in a developing country. Journal of global oncology 2018;4:1-8.
101. Sajadi HS, Ghadirian L, Rajabi F, Sayarifard A, Rostamigooran N, Majdzadeh R. Interventions to increase participation of NGOs in preventive care: A scoping review. Health Science Reports 2022;5(5):e770.
102. Sanadgol A, Doshmangir L, Khodayari-Zarnaq R, Sergeevich Gordeev V. Role of non-governmental organizations in moving toward universal health coverage: A case study in Iran. Frontiers in Public Health 2022;10:985079.
103. Sanadgol A, Doshmangir L, Majdzadeh R, Gordeev VS. Engagement of non-governmental organisations in moving towards universal health coverage: a scoping review. Globalization and health 2021;17(1):1-19.
104. Sanadgol A, Doshmangir L, Majdzadeh R, Gordeev VS. Strategies to strengthen non-governmental organizations' participation in the Iranian health system. Frontiers in Public Health 2022;10:929614.
105. Sankaran S, Ravi PS, Wu YE, Shanabogue S, Ashok S, Agnew K, et al. An NGO-implemented community–clinic health worker approach to providing long-term care for hypertension in a remote region of southern India. Global Health: Science and Practice 2017;5(4):668-77.
106. Sarriot EG, Winch PJ, Ryan LJ, Bowie J, Kouletio M, Swedberg E, et al. A methodological approach and framework for sustainability assessment in NGO□implemented primary health care programs. The International journal of health planning and management 2004;19(1):23-41.
107. Sarwar MR. Bangladesh Health Service Delivery: innovative NGO and private sector partnerships. IDS Bulletin 20.28-17:(3)46;15
108. Shin YA, Yeo J, Jung K. The effectiveness of international non-governmental organizations' response operations during public health emergency: lessons learned from the 2014 Ebola outbreak in Sierra Leone. International Journal of Environmental Research and Public Health 2018;15(4):650.

109. Sikazwe I, Bolton-Moore C, Herce MB. Nongovernmental organizations supporting the HIV service delivery response in Africa–an engine for innovation. Current Opinion in HIV and AIDS 2023;18(1):52-6.
110. Singh MM, Garg S, Nath A, Gupta VK. An assessment of felt needs and expectations of people living with HIV/AIDS seeking treatment at NGOs in Delhi, India. Asia Pacific Journal of Public Health 2015;27(2):NP703-NP12.
111. Sivakumar T, James JW, Basavarajappa C, Parthasarathy R, Kumar CN, Thirthalli J. Impact of community-based rehabilitation for mental illness on 'out of pocket'expenditure in rural South India. Asian journal of psychiatry 2019;44:138-42.
112. Soe KT, Saw S, van Griensven J, Zhou S ,Win L, Chinnakali P, et al. organizations' provision of community-based tuberculosis care for hard-to-reach populations in Myanmar, 2013-2014. Infectious Diseases of Poverty 2017;6:1.
113. Solomon Y, Ballif-Spanvill B, Ward C, Fuhriman A, Widdision-Jones K. The dynamics of community and NGO partnership: primary health care experiences in rural Mali. Promotion & education 2008;15(4):32-7.
114. Srivastava A, Bhattacharyya S, Gautham M, Schellenberg J, Avan BI. Linkages between public and non-government sectors in healthcare: a case study from Uttar Pradesh, India. Global public health 2016;11(10):1216-30.
115. Thomas R, Srinivasan R, Sudarshan H. Nutritional status of tribal children and adolescents in rural south India: the effect of an NGO delivered nutritional programme. The Indian Journal of Pediatrics 2013;80:821-5.
116. Van de Vijver S, Oti S, Cohen Tervaert T, Hankins C, Kyobutungi C, Gomez GB, et al. Introducing a model of cardiovascular prevention in Nairobi's slums by integrating a public health and private-sector approach: the SCALE-UP study. Global health action 2013;6(1):22510.
117. Wamai RG. Reforming health systems: the role of NGOs in Decentralization. Lessons from Kenya and Ethiopia. Baltimore: International society for thirdsector research 2008: 1-21.
118. Wandwalo E, Kapalata N, Tarimo E, Corrigan CB, Morkve O. Collaboration between the national tuberculosis programme and a non governmental organisation in TB/HIV care at a district level: experience from Tanzania. African health sciences 2004; 4(2):109-14.
119. Wang D, Mei G, Xu X, Zhao R, Ma Y, Chen R, et al. Chinese non-governmental organizations involved in HIV/AIDS prevention and control: intra-organizational social capital as a new analytical perspective. Bioscience trends 2016; 10(5):418-23.
120. Yagub AI, Mtshali K. The role of non-governmental organizations in providing curative health services in North Darfur State, Sudan. African health sciences. 2015;15(3):1049-55.
121. Zachariah R, Teck R, Harries A, Humblet P. Implementing joint TB and HIV interventions in a rural district of Malawi: is there a role for an international non-governmental organisation?[unresolved issues]. The international journal of tuberculosis and lung disease 2004;8(9):1058-64.

122. HY MA-H, Ohkado A, Masui T, Ali-Hussein IA, AN SA-A. A trial to mobilize NGO health volunteers to improve tuberculosis patient care in Sana'a City, Yemen. Kekkaku:[Tuberculosis] 2010;85(3):159-62.
123. Matousek AC, Addington SR, Jean-Louis RRE, Pierre JH, Fils J, Hoyler M, et al. The struggle for equity: an examination of surgical services at two NGO hospitals in rural Haiti. The Lancet 2015;385:S20.
124. Mukherjee S, Rashmi C. NGOs role in community–based monitoring of primary health care services for Dalit women in urban slums. Rupkatha J Interdiscip Stud Humanit 2017;9(1):250-62.
125. Singh V, Ahmed S, Dreyfuss ML, Kiran U, Chaudhery DN, Srivastava VK, et al. Non-governmental organization facilitation of a community-based nutrition and health program: Effect on program exposure and associated infant feeding practices in rural India. PLoS One 2017;12(9):e0183316.
126. Babis D. The role of civil society organizations in the institutionalization of indigenous medicine in Bolivia. Social Science & Medicine 2014;123:287-94.
127. Edward A, Branchini C, Aitken I, Roach M, Osei-Bonsu K, Arwal SH. Toward universal coverage in Afghanistan: a multi-stakeholder assessment of capacity investments in the community health worker system. Social science & medicine 2015;145:173-83.
128. Eftekhari MB, Mirabzadeh A, Forouzan AS, Dejman M, Afzali HM, Djalalinia S, et al. A qualitative study of community-based health programs in Iran: an experience of participation in IR Iran. International journal of preventive medicine 2014;5(6):679.
129. Ui S, Heng L, Yatsuya H, Kawaguichi L, Akashi H, Aoyana A. Strengthening community participation at health centers in rural Cambodia: role of local non-governmental organizations (NGOs). Critical Public Health 2010;20(1):97-115.
130. Zihindula G, Andrew John R, Gumede DM, Richard Gavin M. A review on the contributions of NGOs in addressing the shortage of healthcare professionals in rural South Africa. Cogent Social Sciences 2019;5(1):1674100.
131. Sharma A, Singh S, Meena S, Kannan A. Impact of NGO run mid day meal program on nutrition status and growth of primary school children. The Indian Journal of Pediatrics 2010;77:763-9.
132. Chanani S, Waingankar A, Shah More N, Pantvaidya S, Fernandez A, Jayaraman A. Effectiveness of NGO□government partnership to prevent and treat child wasting in urban India. Maternal & child nutrition 2019;15:e12706.
133. Dye-Braumuller KC, Gordon JR, McCoy K, Johnson D, Dinglasan R, Nolan MS. Riding the wave: reactive vector-borne disease policy renders the United States vulnerable to outbreaks and insecticide resistance. Journal of Medical Entomology 2022;59(2):401-11.
134. Welch V, Pottie K, Gaudet C, Thuku M, Mallard R, Spenceley S, et al. Realist review of community coalitions and outreach interventions to increase access to primary care for vulnerable populations: a realist review. Archives of Public Health 2023;81(1):115.

135. Baum F, Freeman T, Sanders D, Labonté R, Lawless A, Javanparast S. Comprehensive primary health care under neo-liberalism in Australia. Social Science & Medicine 2016;168:43-52.
136. Ho AHY, Lall P, Tan WS, Patinadan PV, Wong LH, Dutta O, et al. Sustainable implementation of advance care planning in Asia: An interpretive-systemic framework for national development. Palliative & Supportive Care 2021;19(1):82-92.
137. Ali D, Woldegiorgis AG-Y, Tilaye M, Yilma Y, Berhane HY, Tewahido D, et al. Integrating private health facilities in government-led health systems: a case study of the public–private mix approach in Ethiopia. BMC Health Services Research 2022;22(1):1-16.
138. Zevallos L, Salas V, Robles L. Public-private partnerships for health services: the solution for the peruvian health system? Revista peruana de medicina experimental y salud publica 2014;31(4):769-74.
139. Wallace SP ,Young M-EDT, Rodríguez MA, Brindis CD. A social determinants framework identifying state-level immigrant policies and their influence on health. SSM-population health 2019;7:100316.
140. Rajabi M, Ebrahimi P, Aryankhesal A. Facilitators of improving the function of nongovernmental organizations (NGOs) in Iran's health system. Journal of Education and Health Promotion 2023;12(1):147.
141. Gooding K, Newell JN, Emmel N. Capacity to conduct health research among NGOs in Malawi: Diverse strengths, needs and opportunities for development. PLoS One 2018;13(7):e0198721.
142. Pottie K, Martin JP, Cornish S, Biorklund LM, Gayton I, Doerner F, Schneider F. Access to healthcare for the most vulnerable migrants: a humanitarian crisis. Conflict and health 2015;9(1):1-3.
143. Choudhury MC, Chaube P. Integrating rare disease management in public health programs in India: exploring the potential of National Health Mission. Orphanet Journal of Rare Diseases 2022;17(1):43.
144. Prinja S, Sharma A, Nimesh R, Sharma V ,Madan Gopal K, Badgaiyan N, et al. Impact of National Health Mission on infant mortality in India: An interrupted time series analysis. The International journal of health planning and management 2021;36(4):1143-52.
145. Kabia E, Kazungu J, Barasa E. The effects of health purchasing reforms on equity, access, quality of care, and financial protection in Kenya: a narrative review. Health Systems & Reform 2022;8(2):2114173.
146. Okech TC, Lelegwe SL. Analysis of universal health coverage and equity on health care in Kenya. Global journal of health science 2016;8(7):218.
147. World Health Organization. Sustainable health financing, universal coverage and social health insurance. World Health Assembly Resolution 2005;58:139-40.
148. Carrin G, James C. Reaching universal coverage via social health insurance: key design features in the transition period. Geneva: World Health Organization 2004;13.

149. Jamal MH, Abdul Aziz AF, Aizuddin AN, Aljunid SM. Successes and obstacles in implementing social health insurance in developing and middle-income countries: A scoping review of 5-year recent literatures. Frontiers in Public Health 2022;10:918188.
150. Aspinall E. Health care and democratization in Indonesia. Democratization 2014;21(5):803-23.
151. Wendt C. Social health Insurance in Europe: basic concepts and new principles. Journal of Health Politics, Policy and Law 2019;44(4):665-77.
152. Hartmann C, Hartmann JM, Lopez A, Flores P. Health non-governmental organizations (NGOs) amidst civil unrest: Lessons learned from Nicaragua. Global Public Health 2020;15(12):1810-9.
153. Bawah AA, Phillips JF, Asuming PO, Jackson EF, Walega P, Kanmiki EW, et al. Does the provision of community health services offset the effects of poverty and low maternal educational attainment on childhood mortality? An analysis of the equity effect of the Navrongo experiment in Northern Ghana. SSM-Population Health 2019;7:100335.
154. Reddy JJ, Sharma A, Bhat N, Multani S, Patel R, Singh S. Assessment of health-related activities of non-governmental organizations in Rajasthan, India. The National Medical Journal of India. 2016;29(3):163.
155. Patel V, Beckjord E, Moser RP, Hughes P, Hesse BW. The role of health care experience and consumer information efficacy in shaping privacy and security perceptions of medical records: national consumer survey results. JMIR medical informatics 2015;3(2):e3238.
156. Kabonga I. NGOs and poverty reduction in Zimbabwe: challenges and the way forward. SN Social Sciences 2023;3(6):90.
157. Goma FM. Enhancing Harmonization to Ensure Alignment of Partners, Implementation and Priorities for Provision of Quality Primary Healthcare to Communities in Rural Zambia. World Health & Population 2017;17(3):18-30.
158. Kanazawa R. Positioning AIDS Activism in India: Civil Society Responses Between Development Aid, Global Health and the State, 1989 to 2001. Social History of Medicine 2021;34(1):23-45.
159. Macharia GW. The Role Of Corporate Communication In Local Fundraising In AMREF Health Africa Kenya: School of Communication, Daystar University; 2017.
160. Marimpet TN. Monitoring and Evaluation Practices and Project Performance of Water, Sanitation and Hygiene Projects in Kenya: a Case of the Kenya Red Cross Society: University of Nairobi; 2022.
161. Karkee R, Comfort J. NGOs, foreign aid, and development in Nepal. Frontiers in public health 2016;4:177.

Click here to access/download
Supporting Information
Supplementary file 1.docx

Click here to access/download
Supporting Information
Supplementary file 2.docx

Click here to access/download
Supporting Information
PRISMA-ScR-Fillable-Checklist_10Sept2019.docx
