# Supplementary material for: The involvement of non-governmental organisations in achieving health system goals based on the WHO six building blocks: A scoping review on global evidence
Source: PLoS One. 2025 Jan 30;20(1):e0315592. doi: 10.1371/journal.pone.0315592 (PMC11781716; doi:10.1371/journal.pone.0315592)
Supplement: Attachment — Submitted filename: Point-by-point response.docx [file pone.0315592.s005.docx]

Plos one

25 November 2024

Dear Editor,

Thank you for the comments from you and respected reviewers, which provided us with the opportunity to improve our manuscript. We are pleased to inform you that the authors have had a detailed look at the manuscript and have addressed all comments, as much as possible. The changes to the manuscript were highlighted with the track changes service.

We would hope that you will find these changes up to your satisfaction and look forward to hearing your decision in due course. Please do not hesitate to contact me if any further clarification is required.

Yours truly,

Corresponding author and on behalf of all co-authors,

**Reviewer 1-**

**Topic**: The Involvement of NGOs Operating in Iran? or where?

***Response: This study was conducted in the world literature*.**

**“The involvement of nongovernmental organisations in achieving health system goals based on the WHO six building blocks: A scoping review on global evidence”**

**Introduction:**

In general, I agree with the presentation.

However, also note that: (1) NGOs, Government and Non-state actors, all start from their established interests and positions, and the conclusion you made may not satisfy diverse groups (political, business, advocates, etc.). Therefore, criticisms of government and NGO positions are needed for the author to ensure neutrality.

(2) access to and benefit from health care services by the poor and other diverse groups is both right and privilege, I believe and argue. Thus, a balanced stance, and a kind of disaggregated evidence is needed to ascribe "critical ... etc." place to NGOs.

(3) A push should be on the government to provide health services to all, and NGOs should play a limited aspect of it, via maybe, partnership, and yet, a working partnership between government and NGOs is a contentious area in poor countries where democratic governance barely exist.

I suggest the author position the arguments and substantiate claims from the side of all actors (I mean, authors dwell on positive claims, and silent on critiques). There are repetitions, and the research gaps and inquiry are not presented.

**Response: Thank you for your valuable comments. The introduction, discussion and conclusion parts were revised based on your comments.**

**Methods**

**Screening and selection** –

(1) this is an important part of screening and extracting dependable evidence. I am not convinced.

Also, the screening steps:

(1) using keywords, (2) quickly skimming titles, (3) reading abstracts, (4) reading the whole article/document, and (5) clearing for a systematic scoping review.

**Response: Thank you for your comment. The screening process was revised.**

Based on these steps, how many articles/documents were generated and how many of them were dropped in each step, with justifications are needed in this section.

**Response: Thank you for your comment. The reasons and justifications were provided for deleting studies.**

(2) Assuming you obtained thousands of articles and documents, and some of them might be duplicates. How did the authors manage duplication; for instance, did the authors use EndNote or Mendeley, or what?

**Response:** **We used the Endnote to manage the retrieved studies.**

(3) Data analysis is without theory or model to serve as an analytical lens of interpretation. Since this is a scientific piece of work, the data analysis needs a theoretical/analytical lens. Otherwise, the scoping review report may tend to become something of journalistic work.

**Response: Thank you for your comment. The WHO six building blocks has been used as a model for data analysis.**

(4) The records screed is 1288, yet the records you excluded are 2159. How does the calculation work? Also, why are these records excluded?

**Response: Thank you very much for your valuable comment. The numbers were corrected.**

**Discussion**

A lot of evidence from policy/program/project outcome evaluations shows that collaboration, program coherence and partnership-related objectives are often from those unmet (unachieved)? Besides, these issues are often on paper, and on the ground, not materialized. Thus, I expect the scoping review results to bring concrete evidence on this (including lessons and best practices, if any). Remember my comment on the issue of positioning by government and NGO actors, and how these actors justify their involvement in the health care system (both claim they are effective and serving the people. Yes, how would you show us, maybe, presenting disaggregated evidence along the six building blocks).

**Response: Thank you for your comment. The discussion part was revised based on the comment.**

**Limitations**

The limitations described are bigger than the solutions undertaken. Such a presentation is often counter-productive and questions the findings and the discussions made in the body. Tune down!!

**Response: Thank you for your comment. The limitation part was revised based on the comment.**

**Reviewer #2:**

Title: The Involvement of non-governmental organisations in Achieving Health System Goals Based on the WHO Six Building Blocks: A Scoping Review

**General Overview:**

The manuscript presents a scoping review on the involvement of non-governmental organizations (NGOs) in achieving health system goals, using the WHO’s six building blocks framework as a foundation. The study synthesizes literature from various databases and outlines key strategies that NGOs use to contribute to health system improvements. This review is timely and addresses a significant gap in knowledge regarding the role of NGOs in health system strengthening, particularly in low- and middle-income countries.

**Response: Thank you for reading and commenting on our manuscript.**

**Strengths:**

***Relevance and Importance***: The involvement of NGOs in health system development is a crucial topic, particularly in regions where government resources are limited. The manuscript addresses the diverse ways NGOs contribute to health service delivery, workforce strengthening, and governance, making it highly relevant for policymakers, healthcare planners, and stakeholders in global health.

**Response: Thank you for reading and commenting on our manuscript.**

***Comprehensive Review Methodology***: The use of a scoping review methodology following the JBI framework ensures a broad mapping of available evidence. The review includes multiple databases and uses clear inclusion/exclusion criteria, which enhances the credibility of the findings. The synthesis of results according to the six building blocks is a logical and structured approach, making the data easy to follow and understand.

**Response: Thank you for reading and commenting on our manuscript.**

***Structured Presentation***: The division of results into the six building blocks, along with clear tables outlining strategies and sub-themes, is well-organized. This presentation allows readers to grasp the complexity of NGO involvement in health system strengthening effectively.

**Response: Thank you for reading and commenting on our manuscript.**

***Areas for Improvement****:*

*Lack of Quality Assessment: Although the scoping review methodology does not typically include a formal risk of bias assessment, the manuscript would benefit from some discussion of the quality of the included studies. Since the review covers a wide range of study designs, acknowledging potential limitations in the robustness of the included evidence could add transparency to the findings. Including a brief quality assessment, even if informal, would provide more depth to the discussion.*

**Response: Thank you for the comment. We considered it as a limitation and explained how we considered studies in the synthesis.**

Over-reliance on Qualitative Data: The study synthesizes both qualitative and quantitative data, but the emphasis seems to be on qualitative outcomes, particularly in describing NGO strategies and roles. While this is valuable, there is a lack of quantitative analysis that could further strengthen the conclusions, especially regarding the effectiveness of specific interventions. Including more data on the impact of NGO involvement, in terms of health outcomes or system efficiency, would make the findings more compelling.

**Response: Thank you for your comment. Actually, due to the nature of our study (scoping review), we have focused on the role of NGOs in achieving health system goals and strategies to enhance NGO involvement in achieving health system goals. Assessing the effectiveness and outcomes of specific interventions is therefore outside the scope of our study.**

Vagueness in Some Strategies: While the manuscript identifies several strategies used by NGOs, some of these are too broad or vaguely described. For instance, terms like "strengthening intersectoral coordination" or "promoting links between NGOs" lack specificity in terms of practical implementation. It would be beneficial for the authors to provide more detailed examples or case studies to illustrate how these strategies have been effectively employed in real-world settings.

**Response: Thank for your comment. The strategies were revised based on the comment.**

Geographical Limitations: The review covers a wide range of studies but would benefit from a more explicit discussion of the geographical distribution of the findings. Are certain regions or countries more represented than others? This could introduce biases in the generalizability of the conclusions. A discussion of regional variations in NGO roles or the challenges faced by NGOs in different health systems would enhance the manuscript's depth.

***Response:* Thank you for your comment. The presentation of the findings were revised based on the comment.**

Clarification of Scope: The manuscript occasionally overlaps in its discussion of NGOs versus other private-sector entities. It would be helpful to clearly delineate the distinction between non-profit, NGO, and private-sector involvement in the health system. This clarification would provide more focus and avoid conflating different types of health system actors.

**Response: Thank you for your comment. Was revised.**

**Methodological Clarifications**:

The search strategy is well-documented, but it would be beneficial to explain the choice of 2000 as the starting date for the literature search. Given that NGO involvement in health systems has a longer history, some older studies might still hold relevance.

**Response: Based on the comment, we considered the search without a time limit. However, no relevant studies were found before 2000.**

While the review does not conduct a formal quality assessment, a brief explanation of how study limitations were considered in the synthesis (beyond exclusion criteria) could be helpful for transparency.

**Response: Thank you for your comment. We considered it as a limitation and explained how we considered studies in the synthesis.**

**Conclusion and Recommendations**:

The manuscript provides valuable insights into the role of NGOs in strengthening health systems, but the presentation of the findings could be improved with more specific examples, clearer geographical breakdowns and attention to the quality of the studies included. The discussion is thorough but could be enhanced by focusing on concrete outcomes or quantitative data showing the impact of NGO involvement. Despite these limitations, the review makes a significant contribution to the understanding of NGO roles in health system strengthening and offers actionable strategies for stakeholders looking to enhance NGO involvement.

**Response:** **Thank you for your comment. Was done.**
